# Clinical practice guidelines for the care of people experiencing chronic primary pain: protocol for a systematic review with interpretation against an established chronic pain care priority framework

**DOI:** 10.1136/bmjopen-2025-105315

**Published:** 2025-09-18

**Authors:** Andrew M Briggs, Nandi Siegfried, Robert Waller, Neil E O’Connell, Lorena Romero, Nardia-Rose Klem, Paapa Kwesi Ampiah, Joletta L Belton, Fiona M Blyth, Simone De Morgan, Susan M Lord, Michael Nicholas, Peter B O’Sullivan, Asim Shakya, Anne J Smith, Helen Slater

**Affiliations:** 1 Curtin School of Allied Health, Curtin University, Perth, Western Australia, Australia; 2 Health Systems Research Unit, South African Medical Research Council, Cape Town, South Africa; 3 Independent Clinical Epidemiologist, Cape Town, South Africa; 4 Department of Health Sciences, Brunel University London - Uxbridge Campus, London, UK; 5 Ian Potter Library, Alfred Hospital, Melbourne, Victoria, Australia; 6 Independent Lived Experience Partner, Fraser, Colorado, USA; 7 Sydney School of Public Health, The University of Sydney, Sydney, New South Wales, Australia; 8 Sydney Medical School, The University of Sydney, Sydney, New South Wales, Australia; 9 School of Medicine and Public Health, The University of Newcastle, Newcastle, New South Wales, Australia; 10 The University of Newcastle Hunter Medical Research Institute, New Lambton, New South Wales, Australia; 11 Pain Management Research Institute, The Kolling Institute, The University of Sydney, Sydney, New South Wales, Australia; 12 Independent Lived Experience Partner, Lalitpur, Nepal

**Keywords:** Chronic Pain, Health Equity, Protocols & guidelines, PAIN MANAGEMENT, Patient-Centered Care, Systematic Review

## Abstract

**Abstract:**

**Introduction:**

Most clinical practice guidelines (CPGs) for assessing and managing people’s chronic pain focus on specific pain conditions, body sites or life course stages. This creates complexity for clinicians making care choices in the absence of a diagnosis and/or where a person experiences more than one pain condition. Specific to this context is the ICD-11 classification of *chronic primary pain* where an experience of pain cannot be better accounted for by another condition. CPGs for chronic primary pain, agnostic to condition or body part, may support clinicians towards best pain care since many of the principles of person-centred chronic pain care are transdiagnostic. The two aims of this systematic review are to (1) identify and appraise CPGs for chronic primary pain, relevant across the life course and (2) map the CPG content against a pain care priority framework to evaluate the extent to which the CPG content aligns with the priorities of people with lived chronic pain experience.

**Methods and analysis:**

We will systematically search nine scholarly databases, the Epistemonikos database and international and national guidelines clearinghouses. CPGs published within 2015–2025, in any language, that offer recommendations about assessment and/or management of chronic primary pain for people of any age, excluding hospitalised inpatients or institutionalised populations, will be included. Pairs of reviewers will independently screen citations for eligibility and appraise CPG quality and implementation potential using the Appraisal of Guidelines for Research and Evaluation (AGREE)-II and the AGREE-Recommendations Excellence tools, respectively. Data extraction will include the citation and scope characteristics of each CPG, methods used to develop recommendations, verbatim recommendations, guiding principles or practice information and narrative excerpts related to the GRADE Evidence-to-Decision (EtD) considerations (or equivalent). We will use the PROGRESS-PLUS framework as a checklist to identify whether determinants of health equity were considered by guideline developers. CPG recommendations will be organised according to common topics and categorised in a matrix according to strength and direction. Qualitative content analysis will be used to synthesise excerpts relating to GRADE EtD considerations (or equivalent), and we will map extracted data against an established chronic pain care priority framework to determine the extent to which the CPGs align with values and preferences of people with lived experience. Interpretation will be informed by an interdisciplinary Advisory Group, including lived experience partners.

**Ethics and dissemination:**

Ethical approval is not required for this systematic review. Results will be disseminated through publication in an open-access peer-reviewed journal, through professional societies, and integrated into education curricula and public-facing resources. Reporting will be consistent with the Preferred Reporting Items for Systematic Reviews and Meta-Analysis (PRISMA) statement.

**PROSPERO registration number:**

CRD420251000482.

**STRENGTHS AND LIMITATIONS OF THIS STUDY**

- The clinical practice guideline (CPG) search strategy will be comprehensive, encompassing nine scholarly databases, the Epistemonikos database and guidelines clearinghouses, without restriction on language of publication or life course stage.
- We will extract recommendations and key contextual information from included CPGs, including guiding principles, practice guidance statements and GRADE Evidence-to-Decision (EtD) considerations (or equivalent).
- We will interpret the certainty of recommendations through the parameters of recommendation strength and guideline quality, content-analyse contextual information and map this information against a pain care priority framework that reflects the values and preferences of people living with chronic pain.
- We will harness the expertise and insights of an interdisciplinary Advisory Group, including lived experience partners, to interpret the findings.
- Although we will search across databases and clearinghouses, our searches are limited to databases that can be searched in English and to the period 2015–2025 to align with the chronic primary pain classification for the eleventh International Classification of Diseases (ICD-11), which may exclude earlier CPGs and any CPGs that are indexed with non-English terms only.

## Introduction

 Chronic pain is a leading contributor to the global burden of disease impacting people across the life course.^1^ Although the prevalence of chronic pain varies by age, country, definition and diagnostic group, the prevalence estimates and lived experience burden are consistently high.^1 2^ An analysis of 18 national surveys across 17 countries illustrates this point with 37.3% (CI: 36.7 to 37.8) of adult respondents in developed countries and 41.1% (CI: 40.3 to 41.9) in developing countries reporting a chronic pain condition in the last 12 months (age-standardised), with higher prevalence in females and older adults.^3^ A recent systematic review of European adult populations identified a pooled point prevalence of chronic pain of 21.4% (CI: 18.7 to 24.5).^4^ While more common in older adults,^5^ the experience of chronic pain is also common in children, adolescents and young adults. The most recent estimates from a systematic review of research from 70 countries estimated a pooled point prevalence of chronic pain in children (mean age: 13.4 years) of 20.8% (CI: 19.2 to 22.4).^6^ Another systematic review of studies across 22 countries sampling young adults (15–34 years) reported a pooled prevalence of 11.6% (CI: 9.4 to 14.3).^7^

The experience of chronic pain, irrespective of underlying disease, life course stage, pain type or body site(s), manifests as an enduring burden to individuals, families and communities, as well as to health, social and industrial systems.^1 8 9^ Many people with chronic pain do not have their pain validated as an experience or recognised as a condition in its own right. Access to adequate pain assessment or effective pain care is inconsistent,^10^ particularly for groups such as younger people, older people, minoritised groups or people living in lower socioeconomic circumstances.^11^,^16^ Improving access to holistic models of high-quality pain care requires a long-term system transformation approach.^817^,^19^ Within such an approach, building capacity of the health workforce to provide high-value, person-centred pain care is critical.^18 20 21^ In this context, clinical practice guidelines (CPGs) are one vehicle to strengthen health systems and services to deliver best practice care^22^ and can increase provider satisfaction with care.^23^

Currently, the majority of CPGs for chronic pain are specific to diseases (eg, inflammatory conditions, osteoarthritis and cancer) and body sites (eg, low back pain and neck pain), represent aggregated age groups or are constrained to specific populations, such as middle-aged adults. While this approach may be helpful when reviewing and appraising evidence of interventions by disease, body site or population group, the application to practice may be limited since people experiencing chronic pain commonly present with multiple pain-related and non-pain-related health conditions.^24^,^26^ The corollary is that health professionals often need to navigate multiple clinical guidelines and synthesise layers of evidence, within already constrained resourcing, in order to make sense of what care is needed for whom, with limited guidance on how that care may be delivered to an individual.^27^,^30^

The eleventh International Classification of Diseases (ICD-11) recognises chronic pain as a condition in its own right; the diagnosis of *chronic primary pain* is selected when pain has persisted for more than 3 months, is associated with significant emotional distress and/or functional disability and is not better accounted for by another condition (identified with a specific ICD code).^31^ Since many of the principles of person-centred chronic pain care are transdiagnostic,^132^,^34^ CPGs that address chronic primary pain *in toto* may more helpfully guide clinicians toward holistic, high-value pain care and mitigate pain care silos.

While contemporary best-practice chronic pain care is described as holistic and person-centred,^1^ there is often divergence in what people living with pain value, prefer and prioritise.^9 18^ Aligning pain care approaches with people’s values and preferences is more likely to improve a person’s pain experience, their engagement with care and their well-being. Hence, care recipients’ values and preferences are a key consideration in the GRADE Evidence-to-Decision (EtD) framework for formulating recommendations in CPGs.^35 36^ A recent Australian study derived pain care priorities from the perspectives of people with lived experience and carers (age range: 16–93 years). The framework (‘*Listen to me, learn from me*’) consists of nine priority domains that encapsulate 44 discrete pain care priorities, formulated to shape health professional training efforts in chronic pain care.^18^ This framework offers an opportunity to interrogate the degree to which CPGs for chronic primary pain reflect the derived values and care priorities identified by people living with chronic pain.

The environments and contexts in which people live, work and socialise may also influence their experience of chronic pain and health equity.^1337^,^39^ Further, adverse social circumstances have been cross-sectionally associated with poorer health outcomes in adults experiencing persistent pain,^4 40 41^ while adverse social determinant indicators have been associated with increased likelihood of younger people experiencing chronic pain.^42^ Therefore, consideration of determinants of health equity is relevant to quality pain care, yet it is unclear whether they are considered within CPGs for chronic primary pain, particularly where the GRADE EtD ‘equity’ domain may be one of the least discussed by guideline panels.^36^

The two aims of this systematic review are to (1) identify and appraise (quality, equity and implementation potential) CPGs for chronic primary pain, relevant across the life course and agnostic to underlying disease or body site and (2) map the CPG content against the *Listen to me, learn from me* framework to evaluate the extent to which the CPG content aligns with the priorities of people with lived chronic pain experience.

## Methods and analysis

### Design and reporting

We will undertake a systematic review of CPGs for chronic primary pain with interpretation against established person-centred pain care priorities.^18^ Methods will be consistent with best practice processes^43^ and reported according to the Preferred Reporting Items for Systematic Reviews and Meta-Analysis (PRISMA) 2020 statement.^44^ This protocol for the review was registered on the International Prospective Register of Systematic Reviews (PROSPERO) on 12 May 2025 (CRD420251000482). Searches undertaken on PROSPERO registrations at 1 April 2025 did not identify any overlapping reviews. This protocol is reported according to the Preferred Reporting Items for Systematic Reviews and Meta-Analysis Protocols (PRISMA-P) statement (online supplemental file 1).^45^ The review will initiate on 16 June 2025 and is expected to be completed by 30 September 2026.

### Selection criteria

The ‘Population and clinical indication(s) and condition(s); Intervention(s); Comparator(s), Comparison(s) and key content; Attributes of CPGs; and Recommendation characteristics’ (PICAR) framework was used to frame the review question and define the selection criteria,^43^ as outlined below.

### Population and clinical indication(s) and condition(s)

#### Population

We will include CPGs targeted towards community-dwelling adults (including older people) or children (including adolescents and young adults) experiencing chronic primary pain, irrespective of age, gender or sex or ethnic group. We will not include guidelines targeted towards populations that are hospitalised inpatients or other institutionalised populations (eg, people who are incarcerated). We consider that inpatients and other institutionalised populations represent discrete and homogenous populations with specific care needs. In this context, it would not be clinically meaningful to combine recommendations for care with other (non-institutionalised) populations. Older adults in residential-aged care facilities will be included.

#### Clinical indication/condition

**Figure 1:**
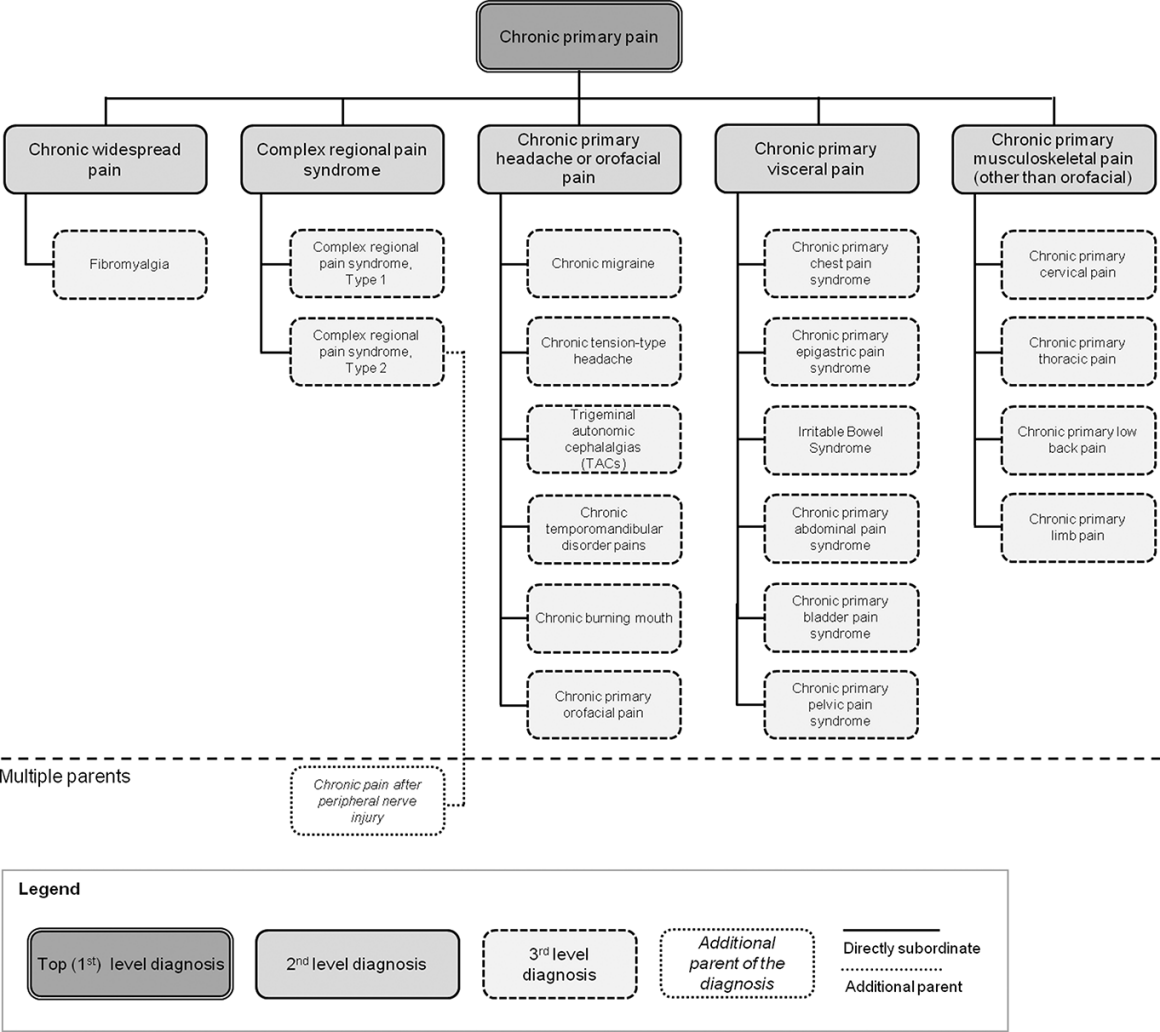
The general structure of the classification of chronic primary pain for the eleventh International Classification of Diseases. Reproduced from Nicholas *et al*^31^ under licence agreement (6026320861589) provided by Wolters Kluwer Health, Inc. and Copyright Clearance Center.

We will include CPGs that offer recommendations on assessment and/or management of chronic primary pain. The term ‘chronic primary pain’ is intended to acknowledge the complex interplay of biological, psychological and social factors that are common to many chronic pain conditions and is agnostic to aetiology/underlying disease (ie, no other condition better explains pain), such as chronic cancer pain, chronic post-surgical or post-traumatic pain, chronic neuropathic pain, chronic secondary headache or orofacial pain, chronic secondary visceral pain or chronic secondary musculoskeletal pain.^31^ Chronic primary pain can manifest in any body system (eg, nervous, musculoskeletal and gastrointestinal systems) and in any body site (face, low back, neck, upper limb, thorax, abdominal, pelvis, urogenital region and lower limb) or in a combination of body sites (eg, widespread pain).^31^ Our inclusion criterion for chronic primary pain will be that the population for which the recommendations are intended within a CPG has experienced pain for more than 3 months and that the pain is not identified as relating to a specific disease or pathology of a body part/site or somatosensory pathway, consistent with the definition of chronic primary pain proposed by International Association for the Study of Pain (IASP) for ICD-11.^31^ CPGs focusing only on a single sub-classification of chronic primary pain (second- or third-level diagnosis (figure 1)) will be excluded, ie, we will not include CPGs with recommendations focused only on chronic widespread pain, complex regional pain syndrome, chronic primary headache or orofacial pain, chronic primary visceral pain or chronic primary musculoskeletal pain or chronic pain specific to a body region (eg, low back pain), organ or underlying condition that is classified as chronic secondary pain. In situations where a CPG contains recommendations for chronic primary pain and recommendations for chronic primary pain sub-classifications or chronic secondary pain conditions, we will extract recommendations for chronic primary pain only.

### Intervention(s)

We will include any recommendation for assessment or management for chronic primary pain undertaken by one or more health professionals of any discipline, including pharmacological, non-pharmacological or other interventions. Recommendations may include single interventions (eg, pharmacotherapy) or integrative interventions, including any delivery mode (group-based, individual, face-to-face or digital). Guidelines focusing on primary prevention of chronic primary pain will be excluded.

### Comparator(s), comparison(s)

We will include any comparator or comparison.

### Attributes of eligible CPGs

We will include all types of CPGs, as defined by the Institute of Medicine (IOM) as *statements that include recommendations, intended to optimise patient care, that are informed by a systematic review of evidence and an assessment of the benefits and harms of alternative care options*.^46^ CPGs may be published in peer-reviewed journals or by professional societies or health agencies. Clinical care standards (including quality standards, care standards and practice standards) and organisation-level position statements that do not meet the IOM definition will be excluded. Personal opinion statements, primary research studies, case reports, protocols and models of care/service delivery will be excluded. We will limit the search period to CPGs published between 2015 and 2025 to ensure a more contemporary evidence base is considered in light of the first call for the ICD-11 classification of chronic primary pain made in 2015.^47^ No restrictions will be placed on publication language or the format of CPGs. In situations where peer-reviewed journal papers provide a summary or excerpt of a CPG, for example, Carville *et al*,^48^ we will use the primary CPG only as the data source. Where multiple versions of the same guideline have been produced (eg, updates or translations), we will use the most recent publication and/or the English language publication only.

### Recommendation characteristics

Only CPGs that include at least one recommendation will be included. Recommendations will be extracted where they provide clear advice or direction on best practice assessment and/or management of chronic primary pain, consistent with the GRADE handbook definition, where recommendations should *answer a focused and sensible healthcare question that leads to an action*. Where statements fit this definition of a recommendation but are not explicitly defined as such, we will extract this text. Any disagreement will be resolved through consensus among the review team.

### Information sources

A detailed search strategy will be prepared by a research librarian (LR) and the multidisciplinary research team. The draft detailed search strategy and Peer Review of Electronic Search Strategies checklist are included in online supplemental file 1.^49^ The search strategy will be adapted to apply to nine scholarly databases (MEDLINE, EMBASE, PsycINFO, AMED, Scopus, Web of Science, CINAHL, Global Health and WHO Global Index Medicus) and the Epistemonikos database.^50^ The WHO Global Index Medicus includes five databases, including the African Index Medicus, Index Medicus for the Eastern Mediterranean Region, Index Medicus for the South-East Asia Region, Latin America and the Caribbean Literature on Health Sciences and the Western Pacific Region Index Medicus. To identify CPGs in the search, we will use a filter adapted from the Canadian Agency for Drugs and Technologies in Health CPG search filter (https://searchfilters.cda-amc.ca), tailored to each database and platform. The detailed search strategies are included in online supplemental file 1. There will be no restrictions on the publication language of CPGs. We will include a grey literature search for CPGs published by clinical societies, national or sub-national governments or United Nations agencies. We will search key national and international CPG clearinghouses (online supplemental file 1). As a supplementary strategy, we will conduct searches in Google and Google Scholar using the keywords ‘guideline’ and ‘chronic pain; chronic primary pain,’ limited to the first 10 pages of results.^51^

We will screen reference lists of included CPGs and perform snowball searches on CPGs published in indexed journals (ie, by reviewing articles that cite an included CPG). An interdisciplinary Advisory Group, comprising experts in the field and lived experience partners, will also be consulted to ensure that all relevant CPGs are identified (refer to the Public and patient involvement section for more details). We will review existing systematic reviews of CPGs for chronic primary pain and screen reference lists of those papers.

### Guideline selection

The yield of citations will be de-duplicated and aggregated into a single Endnote file and imported into Covidence software (Veritas Health Innovation, Melbourne, Australia). Titles and abstracts, and executive summaries for CPGs published as grey literature (where available), will be screened for inclusion eligibility by pairs of clinician-researchers against the PICAR framework. The decision to include or exclude a citation for full text review must be agreed by two reviewers. If a decision cannot be reached through consensus, a third reviewer will arbitrate (AMB or HS). Full text documents will be reviewed by pairs of reviewers according to a standardised process across the review team to determine inclusion or exclusion. Document selection and exclusion will be in a PRISMA flowchart. Where the primary CPG document is supported by technical appendices, these additional documents will be collected and evaluated alongside the primary document.

### Data extraction

Data extraction will be undertaken by one reviewer, using a custom Excel template, and then independently verified by a second reviewer. The extraction template will be piloted on two CPGs and iterated as needed. Any disagreements will be resolved by consensus or arbitration with a third reviewer (AMB or HS). Data extraction and tabulation will include:

- Citation details for each CPG (URL, title, date of publication, authors, publishing agency/developer or journal, country of origin or region, language, accompanying technical documents accessed).
- Scope of the CPG (assessment/diagnosis, management, classes of interventions and number of research (Population, Intervention, Comparator Outcome (PICO)) questions).
- Target CPG users.
- Population characteristics of the intended care recipients.
- Outcomes of interest (health, social and other).
- Target healthcare setting of the CPG, including country and/or jurisdiction.
- CPG currency: the publication date of the CPG and periods covered by the literature search in the guideline, date of revision/planned revision(s).
- Methods used to develop the recommendations, including evidence searched, assessment of certainty of the evidence and methods used to determine the strength and direction of a recommendation.
- Recommendations (verbatim) provided within the CPG (including strength, direction and certainty of the evidence).
- Guiding principles and/or practice statements offered by the CPG authors, where provided.
- GRADE EtD considerations including values and preferences, resource implications, equity, acceptability and feasibility, where provided at the overall guideline level or by recommendation. We will use the PROGRESS-PLUS framework as a checklist to identify whether determinants of health equity were considered within the EtD considerations, or elsewhere in the CPG, to extract information related to equity. Where included CPGs do not apply a GRADE EtD approach, we will map the aligned content to these standard GRADE EtD domains, as appropriate.

Given the nature of the review to extract data from the CPGs as written, we will not contact authors to clarify the content.

### Quality appraisal

Each CPG will be quality-appraised using the Appraisal of Guidelines for Research and Evaluation (AGREE)-II tool.^52^ AGREE-II is designed to assess CPGs developed by local, regional, national or international groups or affiliated governmental organisations and is the most widely used tool internationally for the comprehensive appraisal of CPGs.^53 54^ AGREE-II provides an assessment of the methodological quality of CPGs, informed by 23 items scored on a seven-point Likert scale across six domains, with established construct validity and internal consistency.^55 56^ Pairs of reviewers will each use the AGREE PLUS online appraisal platform when assessing the quality of each included CPG. Where a difference in an item score is higher than two points, the review pairs will discuss the item and reach a consensus decision, with input from a third reviewer, if required. The AGREE PLUS tool collects individual item scores, domain scores (scope and purpose, stakeholder involvement, rigour of development, clarity of presentation, applicability and editorial independence) and overall AGREE-II scores. Reviewers undertaking quality appraisals will have access to standard AGREE-II resources and instructions and attend a briefing session to ensure a standard approach to interpretation of items.

AGREE-II focuses primarily on the methodological quality of a CPG; it does not assess the credibility and implementability of the recommendations within a CPG. The AGREE-Recommendations Excellence (AGREE-REX) tool fills this gap as a usable, reliable and valid tool to evaluate CPG recommendations.^54^ AGREE-REX consists of nine items rated on a seven-point Likert scale organised within three theoretical domains (clinical credibility; stakeholders’ values and preferences; and implementability). AGREE-REX can be applied at the level of individual recommendations, a subset of recommendations, or to the suite of recommendations in a CPG overall. In this review, we will apply AGREE-REX to the suite of recommendations overall. Reviewers will appraise included guidelines with the AGREE-REX tool; the same thresholds for between-reviewer scores will apply, as outlined for AGREE-II.

### Data synthesis and judgement of certainty

Data synthesis and judgement of certainty (where applicable) will be approached in four stages as follows:

- Stage 1: synthesis of CPG recommendations, by the direction and strength of recommendations, within topic areas. Certainty in the evidence will be interpreted by mapping recommendation strength and direction against guideline quality, informed by AGREE-II domain scores.
- Stage 2: analysis and synthesis of specific text excerpts from CPGs (GRADE EtD domains, clinical or good practice guidance statements and guiding principles), where stated, to create categories summarising this content.
- Stage 3: map the CPG content against the *Listen to me, learn from me* framework of nine domains.
- Stage 4: narrative synthesis of findings and recommendations for future CPGs for chronic primary pain.

#### Stage 1: synthesis of CPG recommendations

We will apply a synthesis approach for the CPG recommendations aligned to established methods.^57^,^59^ This will follow a staged four-step process.

1. Organise recommendations into common domains/topics relevant to assessment or management of chronic primary pain. Recommendations with common content or guidance, informed by content analysis, will be organised into conceptually similar domains (eg, measuring pain impact experienced or recommendations around engagement in physical activity). Each unique recommendation will retain links to its source, including the direction, strength and level of certainty as determined by the original CPG development panel.
2. Within domains (step 1), classify recommendations into clinically meaningful implementation groups, based on classification criteria proposed by Lin *et al*^57^ (table 1), which organises recommendations into categories according to the direction and strength of the recommendation(s).
3. Interpret the certainty of the recommendations. As there is currently no gold standard approach to judging the certainty of findings from systematic reviews of CPG recommendations, we will apply a hybrid approach based on methods developed by Kredo *et al.*^60^ Having synthesised the CPG recommendations in steps 1 and 2, for each domain, we will plot each recommendation (as a unique data point) in a matrix by its strength classification (‘should do’ or ‘do not do’; ‘could do’; ‘uncertain’) and AGREE-II scores. In this way, the matrix will provide information about the strength of the recommendation and the quality of the guideline, from which inferences about certainty could be made. For example, recommendations with higher strength and higher quality (right upper quadrant of a matrix) would be interpreted as higher certainty.
4. Develop a narrative synthesis of the information contained in each domain/topic based on a content analysis of the extracted primary data.^61^ This process will be undertaken by one reviewer and presented regularly to the broader review team for discussion and refinement, before seeking input from the Advisory Group. At each stage, an explicit link between the narrative summary and primary data (first-order data) will be retained.

**Table 1:** Definitions for CPG recommendation classification, adapted from Lin *et al*,^57^ under licence agreement (6026240104086) provided by the BMJ Publishing Group Ltd. and Copyright Clearance Center

Classification	Definition	Example terminologies
Should do (strong in favour)	‘Should do’ (strong) recommendations are those that should be applied in all circumstances unless there is a rationale not to. These are based on strong evidence, for example, multiple high-quality studies reporting clinically relevant positive effects, benefits that outweigh risks or when in the opinion of CPG development group members that the benefits are unequivocal.	‘Strong recommendation,’ ‘offer’ and ‘should’ occur.
Could do (conditional in favour or conditional against)	‘Could do’ (conditional) recommendations could be applied depending on the circumstances of individual patients. They are usually based on consistent evidence from multiple lesser quality studies or one high-quality study and where benefits outweigh harms. Note: the ‘could do’ is either in favour or against.	‘Conditional recommendation,’ ‘considered’ ‘may include’ ‘recommend,’ ‘practitioner might’ and ‘suggest.’
Do not do (strong against)	‘Do not do’ recommendations apply when there is strong evidence of no benefit and/or harms outweighing benefits.	‘Do not offer,’ ‘should refrain from,’ ‘do not routinely offer,’ ‘not appropriate’ and ‘should not’.
Uncertain (no recommendation)	‘Uncertain’ applies when there is no recommendation for or against a practice because of incomplete or inconsistent research findings. Not all CPGs provide uncertain recommendations. Note: we will compare the number of recommendations in a CPG against the number of research/PICO questions outlined in the scope of the CPG (where stated) to determine whether there is a discrepancy, which will highlight the likely uncertain/no recommendations.	‘Inconclusive’ or ‘we are unable to recommend for or against,’ ‘inconclusive evidence’ or ‘uncertain.’

CPG, clinical practice guideline; PICO, Population, Intervention, Comparator, Outcome.

#### Stage 2: analysis and synthesis of other text excerpts

For CPG text excerpts other than formal recommendations, including EtD domain excerpts (or equivalent), guiding principles and practice/implementation guidance points, we will use qualitative content analysis to synthesise those data to create categories summarising the content.^62^ We will not judge the certainty of this evidence, since this will most likely be derived from the expert opinion of the CPG development panels. However, the process will be reported according to qualitative content analysis conventions to ensure rigour in the analysis.^62^ The narrated findings from this stage will be shared with the Advisory Group for input.

#### Stage 3: map the CPG content against the *Listen to me, learn from me* framework

We will interpret the synthesised data from stages 1 and 2 by mapping the extracted content against the *Listen to me, learn from me* framework.^18^ We will map two sets of data against the nine categories of the *Listen to me, learn from me* framework. First, we will identify and map which, if any, of the extracted CPG recommendations (from stage 1 analysis) align with one or more of the nine framework categories. Alignment will be determined by considering the verbatim recommendation against the detailed description of each category from the *Listen to me, learn from me* framework (online supplemental file 1). Where alignment between a CPG recommendation and a framework category is equivocal, we will interrogate the CPG recommendation further against the specific granular items underpinning that framework category (ie, there are 44 items underpinning the nine categories of the framework).^18^ Second, we will repeat this process using the findings from the stage 2 analysis in which we synthesise CPG text excerpts, other than formal CPG recommendations. At the end of this mapping process, we will create a grid or heat map (or similar, depending on the nature of the data) that summarises the extent to which person-centred pain care priorities are explicitly reflected in the included CPGs. The mapping outcomes from this stage will be shared with the Advisory Group for input.

#### Stage 4: narrative synthesis, interpretation and recommendations for future CPGs

Once stages 1–3 are completed, we will create an overall narrative synthesis of the findings and provide recommendations, from the perspective of the review team and Advisory Group, to inform future CPGs for chronic primary pain, taking into consideration the scope, quality and certainty of CPG-based recommendations for chronic primary pain and their alignment with priorities for person-centred pain care. Given the expected limited pool of CPGs, we do not plan, *a priori*, to stratify outcomes by CPG quality, consistent with best practice methods.^43^ Depending on the life stages relevant to the included CPGs, we will present findings by life course stage, where possible (eg, children and early adolescents, young people, adults and older people).

### Patient and public involvement

An interdisciplinary Advisory Group will support this review and its reporting. The current composition of the Advisory Group includes people with lived experience of chronic pain (JLB and AS) and clinicians and experts in chronic pain care and classification (FMB, SDM, SML, MN, PBOS and AJS). The Advisory Group will be involved at different phases of the review, including:

- Co-design of the Protocol described in this manuscript;
- Review of the initial yield of CPGs assessed as eligible for inclusion, to determine whether any important CPGs have been missed; and
- Stages 1–4 of the data synthesis. At each of these stages, the outcomes of the synthesis will be shared with the Advisory Group members for their perspectives.

Depending on the nature and scope of the findings, we may invite other members to join the Advisory Group where specific skills or interpretations are needed.

## Ethics and dissemination

Ethical approval is not required for this systematic review. We will report the outcomes in a manuscript prepared for an open-access peer-reviewed journal. We will also disseminate the outcomes via professional societies, such as the IASP, clinical societies and colleges, advocacy organisations and training platforms for health professionals such as the Open Pain Education Network. For people living with pain, we will implement findings, as appropriate, into supportive digital resources such as painHEALTH and youngpainhealth.

## Discussion

Chronic pain is experienced by more than 30% of people globally, representing a major global public health issue.^1 2 63^ Despite this burden and the inequities experienced by people rendered vulnerable,^11^,^16^ access to, and delivery of, quality pain care remains fragmented, particularly in low- and middle-income countries and settings.^64^ In this context, CPGs are one important vehicle to improve care quality, reduce unwarranted care variation and elevate the priority of pain care.^65^ To our knowledge, the proposed systematic review will be the first attempt to identify, appraise and synthesise international CPGs for chronic primary pain across the life course. This will be important to the field to synthesise and appraise existing CPGs and establish a process to update the review as new CPGs are developed.^66^ Previous aligned reviews have focused on chronic musculoskeletal pain and chronic non-cancer pain in primary care settings in adults (search to May 2015; CRD42015022098)^59 67^ and common condition-specific chronic musculoskeletal pain presentations including spinal pain, osteoarthritis and shoulder pain in primary and emergency care settings in adults (search to September 2016; CRD42016051653).^57 68^ These earlier reviews have not evaluated CPGs for chronic primary pain more broadly across the life course and across settings. A currently registered review (CRD42024506022) aims to identify chronic non-cancer pain CPGs relevant to adult care in primary care settings and extract recommendations on de-prescribing/tapering opioids and gabapentinoids; non-opioid and non-pharmacological pain management; and patient-centred communication. This protocol describes searching a narrower selection of databases to December 2023 and reports a narrower scope of guidelines clearinghouses than our protocol. Therefore, the current review will provide novel and contemporary data.

Strengths of the proposed review include exploring a novel approach to interpreting the certainty of CPG recommendations, evaluating the content of included CPGs against an established chronic pain care priority framework^18^ and comprehensively evaluating equity considerations in included CPGs. We will also employ a comprehensive search strategy including ten databases and grey literature sources. An interdisciplinary Advisory Group, including lived experience partners, will support identification of CPGs and interpretation of the data synthesis. In particular, we will harness the wisdom and insights of lived experience partners during interpretation and in narrating knowledge and care guidance gaps. Despite these efforts, we acknowledge that the chronic pain care priority framework was derived from an Australian population and may not be globally transferable in all areas. As a strategy to consider cross-cultural and cross-context considerations in CPG development and CPG alignment with the *Listen to me, learn from me* framework, we intentionally assembled a diverse international review team with representation across high-income and low- and middle-income settings, gender, professional background and life course stage. Further, some relevant CPGs may not be identified due to our reliance on databases that use English language search terms, limiting our search to a 10-year period to align with the introduction of the classification system for chronic primary pain for ICD-11.^47^ We are unlikely to identify recommendations for chronic primary pain care should they be presented or discussed in CPGs that focus on a subclassification of chronic primary pain (eg, low back pain and chronic widespread pain).

## Supplementary material

10.1136/bmjopen-2025-105315 online supplemental file 1
