# Supplementary material for: Clinical practice guidelines for the care of people experiencing chronic primary pain: protocol for a systematic review with interpretation against an established chronic pain care priority framework
Source: BMJ Open. 2025 Sep 18;15(9):e105315. doi: 10.1136/bmjopen-2025-105315 (PMC12458848; doi:10.1136/bmjopen-2025-105315)
Supplement: online supplemental file 1 [file bmjopen-15-9-s001.docx]

Table of Contents

[PRESS Checklist 2](#_Toc206703960)

[Search strategies for scholarly databases 4](#_Toc206703961)

[Ovid Medline 4](#_Toc206703962)

[Embase 6](#_Toc206703963)

[PsycINFO 8](#_Toc206703964)

[AMED 10](#_Toc206703965)

[Scopus 12](#_Toc206703966)

[Web of Science 14](#_Toc206703967)

[CINAHL 16](#_Toc206703968)

[Global Health 18](#_Toc206703969)

[WHO Global Index Medicus 20](#_Toc206703970)

[Epistemonikos 21](#_Toc206703971)

[Guidelines clearinghouses and repositories 23](#_Toc206703972)

[Pain care priority categories of the *“Listen to me, learn from me”* framework 25](#_Toc206703973)

## PRESS Checklist

McGowan J, Sampson M, Salzwedel DM, Cogo E, Foerster V, Lefebvre C. PRESS Peer Review of Electronic Search Strategies: 2015 Guideline Statement. J Clin Epidemiol 2016;75:40-46. DOI: <https://doi.org/10.1016/j.jclinepi.2016.01.021>.

| **Category** | **Items** | **Y / N / not applicable** |
| --- | --- | --- |
| Translation of the research question | Does the search strategy match the research question/PICO? | Y |
|  | Are the search concepts clear? | Y |
|  | Are there too many or too few PICO elements included? | N |
|  | Are the search concepts too narrow or too broad? | N |
|  | Does the search retrieve too many or too few records? (Please show number of hits per line.) | N (see supp file) |
|  | Are unconventional or complex strategies explained? | Not applicable |
| Boolean and proximity operators | Are Boolean or proximity operators used correctly? | Y |
|  | Is the use of nesting with brackets appropriate and effective for the search? | Y |
|  | If NOT is used, is this likely to result in any unintended exclusions? | N |
|  | Could precision be improved by using proximity operators (e.g., adjacent, near, within) or phrase-searching instead of AND? | Not applicable |
|  | Is the width of proximity operators suitable (e.g., might adj5 pick up more variants than adj2)? | Y |
| Subject headings | Are the subject headings relevant? | Y |
|  | Are any relevant subject headings missing; e.g., previous index terms? | N |
|  | Are any subject headings too broad or too narrow? | N |
|  | Are subject headings exploded where necessary and vice versa? | Y |
|  | Are major headings (“starring” or restrict to focus) used? If so, is there adequate justification? | N |
|  | Are subheadings missing? | N |
|  | Are subheadings attached to subject headings? (Floating subheadings may be preferred.) | Not applicable |
|  | Are floating subheadings relevant and used appropriately? | Not applicable |
|  | Are both subject headings and terms in free text (see below) used for each concept? | Y |
| Text word searching | Does the search include all spelling variants in free text (e.g., UK versus US spelling)? | Y |
|  | Does the search include all synonyms or antonyms (e.g., opposites)? | Y |
|  | Does the search capture relevant truncation (i.e., is truncation at the correct place)? | Y |
|  | Is the truncation too broad or too narrow? | N |
|  | Are acronyms or abbreviations used appropriately? Do they capture irrelevant material? Are the full terms also included? | Y |
|  | Are the keywords specific enough or too broad? Are too many or too few keywords used? Are stop words used? | Y |
|  | Have the appropriate fields been searched; e.g., is the choice of the text word fields (.tw.) or all fields (.af.) appropriate? Are there any other fields to be included or excluded (database-specific)? | Y |
|  | Should any long strings be broken into several shorter search statements? | N |
| Spelling, syntax, and line numbers | Are there any spelling errors? | N |
|  | Are there any errors in system syntax; e.g., the use of a truncation symbol from a different search interface? | N |
|  | Are there incorrect line combinations or orphan lines (i.e., lines that are not referred to in the final summation that could indicate an error in an AND or OR statement)? | N |
| Limits and filters | Are all limits and filters used appropriately and are they relevant given the research question? | Y |
|  | Are all limits and filters used appropriately and are they relevant for the database? | Y |
|  | Are any potentially helpful limits or filters missing? Are the limits or filters too broad or too narrow? Can any limits or filters be added or taken away? | N |
|  | Are sources cited for the filters used? | Y |

## Search strategies for scholarly databases

### Ovid Medline

Database(s): **Ovid MEDLINE(R) ALL**1946 to May 16, 2025
Search Strategy:

| **#** | **Searches** | **Results** |
| --- | --- | --- |
| 1 | Chronic Pain/ | 27208 |
| 2 | (chronic primary adj3 pain*).mp. | 197 |
| 3 | (chronic* pain* and primary pain*).mp. | 209 |
| 4 | (chronic* adj2 ("non-specific" or medically unexplained or psychosomatic or undiagnosed or somatoform) adj2 pain*).mp. | 416 |
| 5 | (chronic* adj (nociceptive or nociplastic) adj2 pain*).mp. | 61 |
| 6 | ((prolonged or ongoing or "long standing" or longstanding or enduring or "long lasting" or longlasting or "long-term" or longterm or unrelenting or "un-relenting" or "on-going" or persist*) adj10 primary pain*).mp. | 14 |
| 7 | ((recurrent or constant or sustained or relentless or "drawn-out" or continuous or "ever-present" or "non-remitting" or perpetual or lingering or protracted or episodic) adj20 primary pain*).mp. | 4 |
| 8 | (chronic* pain* and ((recurrent or constant or sustained or relentless or "drawn-out" or continuous or "ever-present" or "non-remitting" or perpetual or lingering or protracted or episodic or prolonged or ongoing or "long standing" or longstanding or enduring or "long lasting" or longlasting or "long-term" or longterm or unrelenting or "un-relenting" or "on-going" or persist*) adj pain)).mp. | 2955 |
| 9 | ((prolonged or ongoing or "long standing" or longstanding or enduring or "long lasting" or longlasting or "long-term" or longterm or unrelenting or "un-relenting" or "on-going" or persist* or continuous or recurrent or constant or sustained or lasting or relentless or "drawn-out" or "ever-present" or "non-remitting" or perpetual or lingering or protracted) adj pain).mp. | 15017 |
| 10 | ((refractory or intractable) adj pain).mp. | 3669 |
| 11 | or/1-10 | 44638 |
| 12 | Critical Pathways/ or Clinical Protocols/ | 38641 |
| 13 | consensus/ or Consensus Development Conference/ or consensus development conferences as topic/ | 39475 |
| 14 | guideline/ or practice guideline/ or guidelines as topic/ or practice guidelines as topic/ | 214482 |
| 15 | Health Planning Guidelines/ | 4169 |
| 16 | Clinical Decision Rules/ | 1021 |
| 17 | (position statement or position paper or policy statement or practice parameter or best practice).ti,ab,kf. | 34524 |
| 18 | (standards or guideline or guidelines).ti,kf. | 152340 |
| 19 | (CPG or CPGs).ti. | 6796 |
| 20 | consensus*.ti,kf. | 41327 |
| 21 | consensus*.ab. /freq=2 | 41109 |
| 22 | ((critical or clinical or practice) adj2 (path? or pathway?)).ti,ab,kf. | 18338 |
| 23 | recommendat*.ti,kf. or guideline recommendation*.ab. | 67684 |
| 24 | (care adj2 (standard or path? or pathway? or map? or plan?)).ti,ab,kf. | 101768 |
| 25 | (algorithm* adj2 (screening or examination or test or tested or testing or assessment* or diagnosis or diagnoses or diagnosed or diagnosing or pharmacotherap* or chemotherap* or chemotreatment* or therap* or treatment* or intervention*)).ti,ab,kf. | 26376 |
| 26 | (guideline* or standards or consensus* or recommendat*).au. | 11 |
| 27 | (algorithm* adj2 (management or care or interdisciplinary care or multidisciplinary care or integrative care or inter-disciplinary care or multi-disciplinary care)).ti,ab,kf. | 4698 |
| 28 | (Guidance statement? or Guidance document? or consensus guidance).ti,ab,kf. | 3057 |
| 29 | ((critical or clinical or practice) adj (model? or service model? or "model? of care")).ti,ab,kf. | 11828 |
| 30 | ((international or national or global or worldwide or world-wide or multinational or multi-national or federal or regional) adj3 guideline*).ab,kf. | 38653 |
| 31 | ((state or provinc*) adj3 guideline*).ab,kf. | 1511 |
| 32 | ((expert panel or consensus or expert group or expert committee or multidisciplinary panel or interdisciplinary panel or specialist panel or expert derived) adj recommendat*).ti,ab,kf. | 4331 |
| 33 | (guideline development group* or guideline development panel*).ti,ab,kf. | 540 |
| 34 | (panel adj (guideline* or guidance or recommendation*)).ti,ab,kf. | 554 |
| 35 | ((WHO or UN or world health or united nations) adj guideline*).ti,ab,kf. | 4829 |
| 36 | or/12-35 | 652601 |
| 37 | 11 and 36 | 2059 |
| 38 | (exp animals/ or exp animal experimentation/ or exp models, animal/ or exp plants/ or exp fungi/) not humans/ | 5807022 |
| 39 | 37 not 38 | 2038 |
| 40 | limit 39 to (autobiography or bibliography or biography or case reports or clinical trial, veterinary or comment or dictionary or legal case or legislation or observational study, veterinary or randomized controlled trial, veterinary) | 90 |
| 41 | 39 not 40 | 1948 |
| 42 | (rat or rats or mouse or mice or swine or porcine or murine or sheep? or lamb or lambs or pig or pigs or piglets or rabbit or rabbits or cat or cats or dog or dogs or cattle or monkey or monkeys or trout or marmoset* or hamster* or primate* or rodent* or Anserine or Aquiline or Assinine or Bovine or Canine or Cervine or Equine or Elaphine or Feline or Hircine or Leporine or Lupine or Murine or Ovine or Porcine or Rusine or Serpentine or Simian or Ursine or Vulpine or Guinea pig or guinea fowl or fish).ti. | 2569898 |
| 43 | 41 not 42 | 1945 |
| **44** | **limit 43 to yr="2015 -Current"** | **1440** |

### Embase

Database(s): **Embase**1947 to 2025 June 04 Search Strategy:

| **#** | **Searches** | **Results** |
| --- | --- | --- |
| 1 | chronic pain/ | 93207 |
| 2 | (chronic primary adj3 pain*).mp. | 229 |
| 3 | (chronic* pain* and primary pain*).mp. | 312 |
| 4 | (chronic* adj2 ("non-specific" or medically unexplained or psychosomatic or undiagnosed or somatoform) adj2 pain*).mp. | 587 |
| 5 | (chronic* adj (nociceptive or nociplastic) adj2 pain*).mp. | 95 |
| 6 | ((prolonged or ongoing or "long standing" or longstanding or enduring or "long lasting" or longlasting or "long-term" or longterm or unrelenting or "un-relenting" or "on-going" or persist*) adj10 primary pain*).mp. | 22 |
| 7 | ((recurrent or constant or sustained or relentless or "drawn-out" or continuous or "ever-present" or "non-remitting" or perpetual or lingering or protracted or episodic) adj20 primary pain*).mp. | 16 |
| 8 | (chronic* pain* and ((recurrent or constant or sustained or relentless or "drawn-out" or continuous or "ever-present" or "non-remitting" or perpetual or lingering or protracted or episodic or prolonged or ongoing or "long standing" or longstanding or enduring or "long lasting" or longlasting or "long-term" or longterm or unrelenting or "un-relenting" or "on-going" or persist*) adj pain)).mp. | 4932 |
| 9 | ((prolonged or ongoing or "long standing" or longstanding or enduring or "long lasting" or longlasting or "long-term" or longterm or unrelenting or "un-relenting" or "on-going" or persist* or continuous or recurrent or constant or sustained or lasting or relentless or "drawn-out" or "ever-present" or "non-remitting" or perpetual or lingering or protracted) adj pain).mp. | 22967 |
| 10 | ((refractory or intractable) adj pain).mp. | 10155 |
| 11 | or/1-10 | 121219 |
| 12 | clinical pathway/ | 11066 |
| 13 | clinical protocol/ | 123920 |
| 14 | consensus development/ or consensus/ | 142509 |
| 15 | practice guideline/ | 607734 |
| 16 | clinical decision rule/ | 1210 |
| 17 | (position statement or position paper or policy statement or practice parameter or best practice).ti,ab,kf. | 50952 |
| 18 | (standards or guideline or guidelines).ti,kf. | 209440 |
| 19 | (CPG or CPGs).ti. | 8226 |
| 20 | consensus*.ti,kf. | 51597 |
| 21 | consensus*.ab. /freq=2 | 55210 |
| 22 | ((critical or clinical or practice) adj2 (path? or pathway?)).ti,ab,kf. | 27695 |
| 23 | recommendat*.ti,kf. or guideline recommendation*.ab. | 87765 |
| 24 | (care adj2 (standard or path? or pathway? or map? or plan?)).ti,ab,kf. | 203272 |
| 25 | (algorithm* adj2 (screening or examination or test or tested or testing or assessment* or diagnosis or diagnoses or diagnosed or diagnosing or pharmacotherap* or chemotherap* or chemotreatment* or therap* or treatment* or intervention*)).ti,ab,kf. | 39223 |
| 26 | (guideline* or standards or consensus* or recommendat*).au,co. | 2445 |
| 27 | (algorithm* adj2 (management or care or interdisciplinary care or multidisciplinary care or integrative care or inter-disciplinary care or multi-disciplinary care)).ti,ab,kf. | 7176 |
| 28 | (Guidance statement? or Guidance document? or consensus guidance).ti,ab,kf. | 4479 |
| 29 | ((critical or clinical or practice) adj (model? or service model? or "model? of care")).ti,ab,kf. | 19078 |
| 30 | ((international or national or global or worldwide or world-wide or multinational or multi-national or federal or regional) adj3 guideline*).ab,kf. | 66295 |
| 31 | ((state or provinc*) adj3 guideline*).ab,kf. | 3478 |
| 32 | ((expert panel or consensus or expert group or expert committee or multidisciplinary panel or interdisciplinary panel or specialist panel or expert derived) adj recommendat*).ti,ab,kf. | 5897 |
| 33 | (guideline development group* or guideline development panel*).ti,ab,kf. | 750 |
| 34 | (panel adj (guideline* or guidance or recommendation*)).ti,ab,kf. | 764 |
| 35 | ((WHO or UN or world health or united nations) adj guideline*).ti,ab,kf. | 7600 |
| 36 | or/12-35 | 1367176 |
| 37 | 11 and 36 | 7911 |
| 38 | (exp animal/ or exp animal experiment/ or exp animal model/ or exp invertebrate/ or exp plant/ or exp fungus/ or nonhuman/) not exp human/ | 9359475 |
| 39 | 37 not 38 | 7808 |
| 40 | limit 39 to (books or chapter or conference abstract or editorial or letter or note) | 2421 |
| 41 | 39 not 40 | 5387 |
| 42 | (rat or rats or mouse or mice or swine or porcine or murine or sheep? or lamb or lambs or pig or pigs or piglets or rabbit or rabbits or cat or cats or dog or dogs or cattle or monkey or monkeys or trout or marmoset* or hamster* or primate* or rodent* or Anserine or Aquiline or Assinine or Bovine or Canine or Cervine or Equine or Elaphine or Feline or Hircine or Leporine or Lupine or Murine or Ovine or Porcine or Rusine or Serpentine or Simian or Ursine or Vulpine or Guinea pig or guinea fowl or fish).ti. | 3219167 |
| 43 | 41 not 42 | 5381 |
| 44 | limit 43 to yr="2015 -Current" | 3257 |
| 45 | 44 not (case report* or autobiography or bibliography or biography or comment or dictionary or legal case or legislation or veterinary).ti. | 3227 |

### PsycINFO

Database(s): **APA PsycInfo**1806 to May 2025 Week 4 Search Strategy:

| **#** | **Searches** | **Results** |
| --- | --- | --- |
| 1 | chronic pain/ | 17935 |
| 2 | (chronic primary adj3 pain*).mp. | 54 |
| 3 | (chronic* pain* and primary pain*).mp. | 90 |
| 4 | (chronic* adj2 ("non-specific" or medically unexplained or psychosomatic or undiagnosed or somatoform) adj2 pain*).mp. | 84 |
| 5 | (chronic* adj (nociceptive or nociplastic) adj2 pain*).mp. | 22 |
| 6 | ((prolonged or ongoing or "long standing" or longstanding or enduring or "long lasting" or longlasting or "long-term" or longterm or unrelenting or "un-relenting" or "on-going" or persist*) adj10 primary pain*).mp. | 4 |
| 7 | ((recurrent or constant or sustained or relentless or "drawn-out" or continuous or "ever-present" or "non-remitting" or perpetual or lingering or protracted or episodic) adj20 primary pain*).mp. | 3 |
| 8 | (chronic* pain* and ((recurrent or constant or sustained or relentless or "drawn-out" or continuous or "ever-present" or "non-remitting" or perpetual or lingering or protracted or episodic or prolonged or ongoing or "long standing" or longstanding or enduring or "long lasting" or longlasting or "long-term" or longterm or unrelenting or "un-relenting" or "on-going" or persist*) adj pain)).mp. | 1278 |
| 9 | ((prolonged or ongoing or "long standing" or longstanding or enduring or "long lasting" or longlasting or "long-term" or longterm or unrelenting or "un-relenting" or "on-going" or persist* or continuous or recurrent or constant or sustained or lasting or relentless or "drawn-out" or "ever-present" or "non-remitting" or perpetual or lingering or protracted) adj pain).mp. | 2957 |
| 10 | ((refractory or intractable) adj pain).mp. | 436 |
| 11 | or/1-10 | 20333 |
| 12 | Treatment Guidelines/ | 10504 |
| 13 | (position statement or position paper or policy statement or practice parameter or best practice).mp. | 10911 |
| 14 | (standards or guideline or guidelines).mp. | 152610 |
| 15 | (CPG or CPGs).ti. | 144 |
| 16 | consensus*.ti,hw. | 4048 |
| 17 | consensus*.ab. /freq=2 | 6587 |
| 18 | ((critical or clinical or practice) adj2 (path? or pathway?)).mp. | 1783 |
| 19 | recommendat*.ti,hw. or guideline recommendation*.ab. | 9813 |
| 20 | (care adj2 (standard or path? or pathway? or map? or plan?)).mp. | 12285 |
| 21 | (algorithm* adj2 (screening or examination or test or tested or testing or assessment* or diagnosis or diagnoses or diagnosed or diagnosing or pharmacotherap* or chemotherap* or chemotreatment* or therap* or treatment* or intervention*)).mp. | 1861 |
| 22 | (guideline* or standards or consensus* or recommendat*).au,ca. | 322 |
| 23 | (algorithm* adj2 (management or care or interdisciplinary care or multidisciplinary care or integrative care or inter-disciplinary care or multi-disciplinary care)).mp. | 238 |
| 24 | (Guidance statement? or Guidance document? or consensus guidance).mp. | 250 |
| 25 | ((critical or clinical or practice) adj (model? or service model? or "model? of care")).mp. | 7243 |
| 26 | ((international or national or global or worldwide or world-wide or multinational or multi-national or federal or regional) adj3 guideline*).ab,hw. | 4048 |
| 27 | ((state or provinc*) adj3 guideline*).ab,hw. | 422 |
| 28 | ((expert panel or consensus or expert group or expert committee or multidisciplinary panel or interdisciplinary panel or specialist panel or expert derived) adj recommendat*).mp. | 304 |
| 29 | (guideline development group* or guideline development panel*).mp. | 69 |
| 30 | (panel adj (guideline* or guidance or recommendation*)).mp. | 70 |
| 31 | ((WHO or UN or world health or united nations) adj guideline*).mp. | 270 |
| 32 | or/12-31 | 195000 |
| 33 | 11 and 32 | 1121 |
| 34 | limit 33 to (bibliography or chapter or "column/opinion" or "comment/reply" or dissertation or editorial or encyclopedia entry or interview or letter or poetry) | 202 |
| 35 | 33 not 34 | 919 |
| 36 | limit 35 to yr="2015 -Current" | 474 |

### AMED

Database(s): **AMED (Allied and Complementary Medicine)**1985 to April 2025 Search Strategy:

| **#** | **Searches** | **Results** |
| --- | --- | --- |
| 1 | (chronic primary adj3 pain*).mp. | 5 |
| 2 | (chronic* pain* and primary pain*).mp. | 6 |
| 3 | (chronic* adj2 ("non-specific" or medically unexplained or psychosomatic or undiagnosed or somatoform) adj2 pain*).mp. | 48 |
| 4 | (chronic* adj (nociceptive or nociplastic) adj2 pain*).mp. | 3 |
| 5 | ((prolonged or ongoing or "long standing" or longstanding or enduring or "long lasting" or longlasting or "long-term" or longterm or unrelenting or "un-relenting" or "on-going" or persist*) adj10 primary pain*).mp. | 0 |
| 6 | ((recurrent or constant or sustained or relentless or "drawn-out" or continuous or "ever-present" or "non-remitting" or perpetual or lingering or protracted or episodic) adj20 primary pain*).mp. | 0 |
| 7 | (chronic* pain* and ((recurrent or constant or sustained or relentless or "drawn-out" or continuous or "ever-present" or "non-remitting" or perpetual or lingering or protracted or episodic or prolonged or ongoing or "long standing" or longstanding or enduring or "long lasting" or longlasting or "long-term" or longterm or unrelenting or "un-relenting" or "on-going" or persist*) adj pain)).mp. | 55 |
| 8 | ((prolonged or ongoing or "long standing" or longstanding or enduring or "long lasting" or longlasting or "long-term" or longterm or unrelenting or "un-relenting" or "on-going" or persist* or continuous or recurrent or constant or sustained or lasting or relentless or "drawn-out" or "ever-present" or "non-remitting" or perpetual or lingering or protracted) adj pain).mp. | 511 |
| 9 | ((refractory or intractable) adj pain).mp. | 114 |
| 10 | or/1-9 | 681 |
| 11 | (position statement or position paper or policy statement or practice parameter or best practice).mp. | 684 |
| 12 | (standards or guideline or guidelines).mp. | 26615 |
| 13 | (CPG or CPGs).mp. | 109 |
| 14 | consensus*.ti,hw. | 379 |
| 15 | consensus*.ab. /freq=2 | 446 |
| 16 | ((critical or clinical or practice) adj2 (path? or pathway?)).mp. | 178 |
| 17 | recommendat*.ti,hw. or guideline recommendation*.ab. | 780 |
| 18 | (care adj2 (standard or path? or pathway? or map? or plan?)).mp. | 1563 |
| 19 | (algorithm* adj2 (screening or examination or test or tested or testing or assessment* or diagnosis or diagnoses or diagnosed or diagnosing or pharmacotherap* or chemotherap* or chemotreatment* or therap* or treatment* or intervention*)).mp. | 215 |
| 20 | (guideline* or standards or consensus* or recommendat*).au. | 1 |
| 21 | (algorithm* adj2 (management or care or interdisciplinary care or multidisciplinary care or integrative care or inter-disciplinary care or multi-disciplinary care)).mp. | 41 |
| 22 | (Guidance statement? or Guidance document? or consensus guidance).mp. | 13 |
| 23 | ((critical or clinical or practice) adj (model? or service model? or "model? of care")).mp. | 172 |
| 24 | ((international or national or global or worldwide or world-wide or multinational or multi-national or federal or regional) adj3 guideline*).ab,hw. | 357 |
| 25 | ((state or provinc*) adj3 guideline*).ab,hw. | 23 |
| 26 | ((expert panel or consensus or expert group or expert committee or multidisciplinary panel or interdisciplinary panel or specialist panel or expert derived) adj recommendat*).mp. | 37 |
| 27 | (guideline development group* or guideline development panel*).mp. | 6 |
| 28 | (panel adj (guideline* or guidance or recommendation*)).mp. | 7 |
| 29 | ((WHO or UN or world health or united nations) adj guideline*).mp. | 19 |
| 30 | or/11-29 | 29568 |
| 31 | 10 and 30 | 56 |
| 32 | limit 31 to yr="2015 -Current" | 20 |

### Scopus

( ( ( TITLE-ABS-KEY ( ( "chronic primary" W/2 pain* ) ) OR TITLE-ABS-KEY ( ( "chronic* pain*" AND "primary pain*" ) ) OR TITLE-ABS-KEY ( ( chronic* W/11 ( "non-specific" OR "medically unexplained" OR psychosomatic OR undiagnosed OR somatoform ) W/1 pain* ) ) OR TITLE-ABS-KEY ( ( chronic* W/0 ( nociceptive OR nociplastic ) W/1 pain* ) ) OR TITLE-ABS-KEY ( ( ( prolonged OR ongoing OR "long standing" OR longstanding OR enduring OR "long lasting" OR longlasting OR "long-term" OR longterm OR unrelenting OR "un-relenting" OR "on-going" OR persist* ) W/9 "primary pain*" ) ) OR TITLE-ABS-KEY ( ( ( recurrent OR constant OR sustained OR relentless OR "drawn-out" OR continuous OR "ever-present" OR "non-remitting" OR perpetual OR lingering OR protracted OR episodic ) W/20 "primary pain*" ) ) ) ) OR ( ( TITLE-ABS-KEY ( ( "chronic* pain*" AND ( ( recurrent OR constant OR sustained OR relentless OR "drawn-out" OR continuous OR "ever-present" OR "non-remitting" OR perpetual OR lingering OR protracted OR episodic OR prolonged OR ongoing OR "long standing" OR longstanding OR enduring OR "long lasting" OR longlasting OR "long-term" OR longterm OR unrelenting OR "un-relenting" OR "on-going" OR persist* ) W/0 pain ) ) ) OR TITLE-ABS-KEY ( ( ( prolonged OR ongoing OR "long standing" OR longstanding OR enduring OR "long lasting" OR longlasting OR "long-term" OR longterm OR unrelenting OR "un-relenting" OR "on-going" OR persist* OR continuous OR recurrent OR constant OR sustained OR lasting OR relentless OR "drawn-out" OR "ever-present" OR "non-remitting" OR perpetual OR lingering OR protracted ) W/0 pain ) ) ) ) ) AND ( ( ( TITLE-ABS-KEY ( ( "position statement" OR "position paper" OR "policy statement" OR "practice parameter" OR "best practice" ) ) OR TITLE ( ( standards OR guideline OR guidelines ) ) OR TITLE ( ( cpg OR cpgs ) ) OR TITLE ( ( consensus* OR recommendat* ) ) OR ABS ( ( consensus* OR "guideline recommendation*" ) ) OR TITLE-ABS-KEY ( ( ( critical OR clinical OR practice ) W/1 ( path OR pathway ) ) ) OR TITLE-ABS-KEY ( ( care W/1 ( standard OR path OR pathway OR map OR plan ) ) ) OR TITLE-ABS-KEY ( ( algorithm* W/1 ( screening OR examination OR test OR tested OR testing OR assessment* OR diagnosis OR diagnoses OR diagnosed OR diagnosing OR pharmacotherap* OR chemotherap* OR chemotreatment* OR therap* OR treatment* OR intervention* ) ) ) ) ) OR ( ( TITLE-ABS-KEY ( ( algorithm* W/1 ( management OR care OR "interdisciplinary care" OR "multidisciplinary care" OR "integrative care" OR "inter-disciplinary care" OR "multi-disciplinary care" ) ) ) OR TITLE-ABS-KEY ( ( "Guidance statement*" OR "Guidance document*" OR "consensus guidance" ) ) OR TITLE-ABS-KEY ( ( ( critical OR clinical OR practice ) W/0 ( model OR "service model*" OR "model* of care" ) ) ) OR TITLE-ABS-KEY ( ( ( international OR national OR global OR worldwide OR "world-wide" OR multinational OR "multi-national" OR federal OR regional ) W/2 guideline* ) ) OR TITLE-ABS-KEY ( ( ( state OR provinc* ) W/2 guideline* ) ) OR TITLE-ABS-KEY ( ( ( "expert panel" OR consensus OR "expert group" OR "expert committee" OR "multidisciplinary panel" OR "interdisciplinary panel" OR "specialist panel" OR "expert derived" ) W/0 recommendat* ) ) OR TITLE-ABS-KEY ( ( "guideline development group*" OR "guideline development panel*" ) ) OR TITLE-ABS-KEY ( ( panel W/0 ( guideline* OR guidance OR recommendation* ) ) ) OR TITLE-ABS-KEY ( ( ( "WHO" OR "UN" OR "world health" OR "united nations" ) W/0 guideline* ) ) ) ) )

**EQUALS 802 RESULTS**

AND LIMIT TO PUBYEAR > 2014 AND PUBYEAR < 2026

**EQUALS 514 RESULTS**

AND ( EXCLUDE ( DOCTYPE , "ch" ) OR EXCLUDE ( DOCTYPE , "ed" ) OR EXCLUDE ( DOCTYPE , "le" ) OR EXCLUDE ( DOCTYPE , "bk" ) )

**EQUALS 495 RESULTS**

### Web of Science

1. ( ( TS =( ( "chronic primary" NEAR/2 pain* ) ) OR TS=( ( "chronic* pain*" AND "primary pain*" ) ) OR TS=( ( chronic* NEAR/11 ( "non-specific" OR "medically unexplained" OR psychosomatic OR undiagnosed OR somatoform ) NEAR/1 pain* ) ) OR TS=( ( chronic* NEAR/0 ( nociceptive OR nociplastic ) NEAR/1 pain* ) ) OR TS=( ( ( prolonged OR ongoing OR "long standing" OR longstanding OR enduring OR "long lasting" OR longlasting OR "long-term" OR longterm OR unrelenting OR "un-relenting" OR "on-going" OR persist* ) NEAR/9 "primary pain*" ) ) OR TS=( ( ( recurrent OR constant OR sustained OR relentless OR "drawn-out" OR continuous OR "ever-present" OR "non-remitting" OR perpetual OR lingering OR protracted OR episodic ) NEAR/20 "primary pain*" ) ) ) ) OR ( ( TS=( ( "chronic* pain*" AND ( ( recurrent OR constant OR sustained OR relentless OR "drawn-out" OR continuous OR "ever-present" OR "non-remitting" OR perpetual OR lingering OR protracted OR episodic OR prolonged OR ongoing OR "long standing" OR longstanding OR enduring OR "long lasting" OR longlasting OR "long-term" OR longterm OR unrelenting OR "un-relenting" OR "on-going" OR persist* ) NEAR/0 pain ) ) ) OR TS=( ( ( prolonged OR ongoing OR "long standing" OR longstanding OR enduring OR "long lasting" OR longlasting OR "long-term" OR longterm OR unrelenting OR "un-relenting" OR "on-going" OR persist* OR continuous OR recurrent OR constant OR sustained OR lasting OR relentless OR "drawn-out" OR "ever-present" OR "non-remitting" OR perpetual OR lingering OR protracted ) NEAR/0 pain ) ) ) )

2. ( TS=( ( "position statement" OR "position paper" OR "policy statement" OR "practice parameter" OR "best practice" ) ) OR TI=( ( standards OR guideline OR guidelines ) ) OR TI=( ( cpg OR cpgs ) ) OR TI=( ( consensus* OR recommendat* ) ) OR AB=( ( consensus* OR "guideline recommendation*" ) ) OR TS=( ( ( critical OR clinical OR practice ) NEAR/1 ( path? OR pathway? ) ) ) OR TS=( ( care NEAR/1 ( standard OR path OR pathway OR map OR plan ) ) ) OR TS=( ( algorithm* NEAR/1 ( screening OR examination OR test OR tested OR testing OR assessment* OR diagnosis OR diagnoses OR diagnosed OR diagnosing OR pharmacotherap* OR chemotherap* OR chemotreatment* OR therap* OR treatment* OR intervention* ) ) ) )

3. ( TS=( ( algorithm* NEAR/1 ( management OR care OR "interdisciplinary care" OR "multidisciplinary care" OR "integrative care" OR "inter-disciplinary care" OR "multi-disciplinary care" ) ) ) OR TS=( ( "Guidance statement*" OR "Guidance document*" OR "consensus guidance" ) ) OR TS=( ( ( critical OR clinical OR practice ) NEAR/0 ( model OR "service model*" OR "model of care" ) ) ) OR TS=( ( ( international OR national OR global OR worldwide OR "world-wide" OR multinational OR "multi-national" OR federal OR regional ) NEAR/2 guideline* ) ) OR TS= ( ( ( state OR provinc* ) NEAR/2 guideline* ) ) OR TS=( ( ( "expert panel" OR consensus OR "expert group" OR "expert committee" OR "multidisciplinary panel" OR "interdisciplinary panel" OR "specialist panel" OR "expert derived" ) NEAR/0 recommendat* ) ) OR TS=( ( "guideline development group*" OR "guideline development panel*" ) ) OR TS=( ( panel NEAR/0 ( guideline* OR guidance OR recommendation* ) ) ) OR TS=( ( ( "WHO" OR "UN" OR "world health" OR "united nations" ) NEAR/0 guideline* ) ) )

4. 2 OR 3

5. 1 AND 4

**EQUALS 802 RESULTS**

AND Refined By: Publication Years: 2025 or 2024 or 2023 or 2022 or 2021 or 2020 or 2019 or 2018 or 2017 or 2016 or 2015.

**EQUALS 506 RESULTS**

AND NOT Document Types: Editorial Material or Meeting Abstract.

**EQUALS 485 RESULTS**

### CINAHL

| **#** | **Query** | **Results** |
| --- | --- | --- |
| S1 | (MH "Chronic Pain") | 28,399 |
| S2 | ("chronic primary" N2 pain*) | 86 |
| S3 | ("chronic* pain*" and "primary pain*") | 80 |
| S4 | (chronic* N1 ("non-specific" or "medically unexplained" or psychosomatic or undiagnosed or somatoform) N1 pain*) | 205 |
| S5 | (chronic* N0 (nociceptive or nociplastic) N1 pain*) | 15 |
| S6 | ((prolonged or ongoing or "long standing" or longstanding or enduring or "long lasting" or longlasting or "long-term" or longterm or unrelenting or "un-relenting" or "on-going" or persist*) N9 "primary pain*") | 3 |
| S7 | ((recurrent or constant or sustained or relentless or "drawn-out" or continuous or "ever-present" or "non-remitting" or perpetual or lingering or protracted or episodic) N20 "primary pain*") | 152 |
| S8 | ("chronic* pain*" and ((recurrent or constant or sustained or relentless or "drawn-out" or continuous or "ever-present" or "non-remitting" or perpetual or lingering or protracted or episodic or prolonged or ongoing or "long standing" or longstanding or enduring or "long lasting" or longlasting or "long-term" or longterm or unrelenting or "un-relenting" or "on-going" or persist*) N0 pain)) | 1,496 |
| S9 | ((prolonged or ongoing or "long standing" or longstanding or enduring or "long lasting" or longlasting or "long-term" or longterm or unrelenting or "un-relenting" or "on-going" or persist* or continuous or recurrent or constant or sustained or lasting or relentless or "drawn-out" or "ever-present" or "non-remitting" or perpetual or lingering or protracted) N0 pain) | 6,133 |
| S10 | ((refractory or intractable) N0 pain) | 993 |
| S11 | S1 OR S2 OR S3 OR S4 OR S5 OR S6 OR S7 OR S8 OR S9 OR S10 | 34,304 |
| S12 | (MH "Critical Path") | 6,338 |
| S13 | (MH "Protocols") OR (MH "Nursing Protocols") | 28,461 |
| S14 | (MH "Consensus") | 9,652 |
| S15 | (MH "Practice Guidelines") | 89,893 |
| S16 | ("position statement" or "position paper" or "policy statement" or "practice parameter" or "best practice") | 20,375 |
| S17 | TI ((standards or guideline or guidelines)) | 89,819 |
| S18 | TI ((CPG or CPGs)) | 445 |
| S19 | XB consensus* | 60,161 |
| S20 | ((critical or clinical or practice) N1 (path# or pathway#)) | 9,654 |
| S21 | TI recommendat* OR AB “guideline recommendation*” | 27,816 |
| S22 | (care N1 (standard or path# or pathway# or map# or plan#)) | 85,846 |
| S23 | (algorithm* N1 (screening or examination or test or tested or testing or assessment* or diagnosis or diagnoses or diagnosed or diagnosing or pharmacotherap* or chemotherap* or chemotreatment* or therap* or treatment* or intervention*)) | 5,991 |
| S24 | CA ((guideline* or standards or consensus* or recommendat*)) | 1,705 |
| S25 | (algorithm* N1 (management or care or "interdisciplinary care" or "multidisciplinary care" or "integrative care" or "inter-disciplinary care" or "multi-disciplinary care")) | 1,404 |
| S26 | ("Guidance statement*" or "Guidance document*" or "consensus guidance") | 970 |
| S27 | ((critical or clinical or practice) N0 (model# or "service model*" or "model? of care")) | 4,978 |
| S28 | ((international or national or global or worldwide or "world-wide" or multinational or "multi-national" or federal or regional) N2 guideline*) | 14,498 |
| S29 | ((state or provinc*) N2 guideline*) | 1,091 |
| S30 | (("expert panel" or consensus or "expert group" or "expert committee" or "multidisciplinary panel" or "interdisciplinary panel" or "specialist panel" or "expert derived") N0 recommendat*) | 1,435 |
| S31 | ("guideline development group*" or "guideline development panel*") | 233 |
| S32 | (panel N0 (guideline* or guidance or recommendation*)) | 532 |
| S33 | ((WHO or UN or "world health" or "united nations") N0 guideline*) | 1,189 |
| S34 | S12 OR S13 OR S14 OR S15 OR S16 OR S17 OR S18 OR S19 OR S20 OR S21 OR S22 OR S23 OR S24 OR S25 OR S26 OR S27 OR S28 OR S29 OR S30 OR S31 OR S32 OR S33 | 369,135 |
| S35 | S11 AND S34 | 2,106 |
| S36 | ( (MH "Animals+") OR (MH "Animal Studies") OR (MH "Animal Diseases+") OR (MH "Birds+") OR (MH "Mammals") OR (MH "Rodents+") OR (MH "Invertebrates+") OR (MH "Animal Population Groups+") OR (MH "Reptiles+") OR (MH "Plants+") ) NOT (MH "Human") | 294,570 |
| S37 | S35 NOT S36 | 2,059 |
| S38 | PT (Anecdote or Audiovisual or Bibliography or Biography or Book or "Book chapter" or Commentary or Directories or "Doctoral dissertation" or Editorial or "Legal cases" or Letter or "Masters thesis" or Pamphlet or Pictorial) | 1,992,055 |
| S39 | S37 NOT S38 | 1,698 |
| S40 | S37 NOT S38  Limiters - Publication Date: 20150101-20251231 | 1,037 |

### Global Health

Database(s): **Global Health**1910 to 2025 Week 23 Search Strategy:

| **#** | **Searches** | **Results** |
| --- | --- | --- |
| 1 | (chronic primary adj3 pain*).mp. | 3 |
| 2 | (chronic* pain* and primary pain*).mp. | 6 |
| 3 | (chronic* adj2 ("non-specific" or medically unexplained or psychosomatic or undiagnosed or somatoform) adj2 pain*).mp. | 23 |
| 4 | (chronic* adj (nociceptive or nociplastic) adj2 pain*).mp. | 2 |
| 5 | ((prolonged or ongoing or "long standing" or longstanding or enduring or "long lasting" or longlasting or "long-term" or longterm or unrelenting or "un-relenting" or "on-going" or persist*) adj10 primary pain*).mp. | 0 |
| 6 | ((recurrent or constant or sustained or relentless or "drawn-out" or continuous or "ever-present" or "non-remitting" or perpetual or lingering or protracted or episodic) adj20 primary pain*).mp. | 0 |
| 7 | (chronic* pain* and ((recurrent or constant or sustained or relentless or "drawn-out" or continuous or "ever-present" or "non-remitting" or perpetual or lingering or protracted or episodic or prolonged or ongoing or "long standing" or longstanding or enduring or "long lasting" or longlasting or "long-term" or longterm or unrelenting or "un-relenting" or "on-going" or persist*) adj pain)).mp. | 62 |
| 8 | ((prolonged or ongoing or "long standing" or longstanding or enduring or "long lasting" or longlasting or "long-term" or longterm or unrelenting or "un-relenting" or "on-going" or persist* or continuous or recurrent or constant or sustained or lasting or relentless or "drawn-out" or "ever-present" or "non-remitting" or perpetual or lingering or protracted) adj pain).mp. | 593 |
| 9 | ((refractory or intractable) adj pain).mp. | 57 |
| 10 | or/1-9 | 680 |
| 11 | guidelines/ | 90760 |
| 12 | (position statement or position paper or policy statement or practice parameter or best practice).mp. | 5655 |
| 13 | (standards or guideline or guidelines).mp. | 247412 |
| 14 | (CPG or CPGs).ti. | 697 |
| 15 | consensus*.ti,hw. | 3745 |
| 16 | consensus*.ab. /freq=2 | 4699 |
| 17 | ((critical or clinical or practice) adj2 (path? or pathway?)).mp. | 1934 |
| 18 | recommendat*.ti,hw. or guideline recommendation*.ab. | 95444 |
| 19 | (care adj2 (standard or path? or pathway? or map? or plan?)).mp. | 12848 |
| 20 | (algorithm* adj2 (screening or examination or test or tested or testing or assessment* or diagnosis or diagnoses or diagnosed or diagnosing or pharmacotherap* or chemotherap* or chemotreatment* or therap* or treatment* or intervention*)).mp. | 3444 |
| 21 | (guideline* or standards or consensus* or recommendat*).au. | 0 |
| 22 | (algorithm* adj2 (management or care or interdisciplinary care or multidisciplinary care or integrative care or inter-disciplinary care or multi-disciplinary care)).mp. | 423 |
| 23 | (Guidance statement? or Guidance document? or consensus guidance).mp. | 969 |
| 24 | ((critical or clinical or practice) adj (model? or service model? or "model? of care")).mp. | 909 |
| 25 | ((international or national or global or worldwide or world-wide or multinational or multi-national or federal or regional) adj3 guideline*).ab,hw. | 11857 |
| 26 | ((state or provinc*) adj3 guideline*).ab,hw. | 353 |
| 27 | ((expert panel or consensus or expert group or expert committee or multidisciplinary panel or interdisciplinary panel or specialist panel or expert derived) adj recommendat*).mp. | 448 |
| 28 | (guideline development group* or guideline development panel*).mp. | 103 |
| 29 | (panel adj (guideline* or guidance or recommendation*)).mp. | 74 |
| 30 | ((WHO or UN or world health or united nations) adj guideline*).mp. | 4807 |
| 31 | or/11-30 | 276839 |
| 32 | 10 and 31 | 42 |
| 33 | limit 32 to yr="2015 -Current" | 25 |

### WHO Global Index Medicus

"chronic primary pain" OR "chronic pain" OR "non-specific pain" OR "medically unexplained pain" OR "psychosomatic pain" OR "undiagnosed pain" OR "somatoform pain" OR "chronic nociceptive pain" OR "chronic nociplastic pain" OR “prolonged primary pain” OR “ongoing primary pain” OR "long standing primary pain" OR “longstanding primary pain” OR “enduring primary pain” OR "long lasting primary pain" OR “longlasting primary pain” OR "long-term primary pain" OR “longterm primary pain” OR “unrelenting primary pain” OR "un-relenting primary pain" OR "on-going primary pain" OR “persistent primary pain” or “persisting primary pain” OR “recurrent primary pain” OR “constant primary pain” OR “sustained primary pain” OR “relentless primary pain” OR "drawn-out primary pain” OR “continuous primary pain”

OR "ever-present primary pain” OR "non-remitting primary pain” OR “perpetual primary pain” OR “lingering primary pain” OR “protracted primary pain” OR “episodic primary pain*"

**AND**

"position statement" OR "position paper" OR "policy statement" OR "practice parameter" OR "best practice" OR standards OR guideline OR guidelines OR cpg OR cpgs OR consensus OR recommendations OR “critical path” OR “clinical path” OR “practice path” OR “critical pathway” OR “clinical pathway” OR “practice pathway” OR algorithm OR "Guidance statement" OR "Guidance document" OR “Critical model” OR “clinical model” OR “practice model” OR "critical service model*" OR "clinical service model*" OR “practice service model” OR "model of care"

EQUALS **261 RESULTS**

LIMITED TO 2015 TO 2025 EQUALS **171 RESULTS**

**These 171 results consist of the following:**

LILACS (Americas) (120)

WPRIM (Western Pacific) (41)

IMSEAR (South-East Asia) (5)

AIM (Africa) (3)

IMEMR (Eastern Mediterranean) (2)

### Epistemonikos

(title:((title:("chronic primary pain" OR "chronic pain" OR "non-specific pain" OR "medically unexplained pain" OR "psychosomatic pain" OR "undiagnosed pain" OR "somatoform pain" OR "chronic nociceptive pain" OR "chronic nociplastic pain" OR "prolonged primary pain" OR "ongoing primary pain" OR "long standing primary pain" OR "longstanding primary pain" OR "enduring primary pain" OR "long lasting primary pain" OR "longlasting primary pain" OR "long-term primary pain" OR "longterm primary pain" OR "unrelenting primary pain" OR "un-relenting primary pain" OR "on-going primary pain" OR "persistent primary pain" OR "persisting primary pain" OR "recurrent primary pain" OR "constant primary pain" OR "sustained primary pain" OR "relentless primary pain" OR "drawn-out primary pain" OR "continuous primary pain" OR "ever-present primary pain" OR "non-remitting primary pain" OR "perpetual primary pain" OR "lingering primary pain" OR "protracted primary pain" OR "episodic primary pain*") OR abstract:("chronic primary pain" OR "chronic pain" OR "non-specific pain" OR "medically unexplained pain" OR "psychosomatic pain" OR "undiagnosed pain" OR "somatoform pain" OR "chronic nociceptive pain" OR "chronic nociplastic pain" OR "prolonged primary pain" OR "ongoing primary pain" OR "long standing primary pain" OR "longstanding primary pain" OR "enduring primary pain" OR "long lasting primary pain" OR "longlasting primary pain" OR "long-term primary pain" OR "longterm primary pain" OR "unrelenting primary pain" OR "un-relenting primary pain" OR "on-going primary pain" OR "persistent primary pain" OR "persisting primary pain" OR "recurrent primary pain" OR "constant primary pain" OR "sustained primary pain" OR "relentless primary pain" OR "drawn-out primary pain" OR "continuous primary pain" OR "ever-present primary pain" OR "non-remitting primary pain" OR "perpetual primary pain" OR "lingering primary pain" OR "protracted primary pain" OR "episodic primary pain*")) AND (title:("position statement" OR "position paper" OR "policy statement" OR "practice parameter" OR "best practice" OR standards OR guideline OR guidelines OR cpg OR cpgs OR consensus OR recommendations OR "critical path" OR "clinical path" OR "practice path" OR "critical pathway" OR "clinical pathway" OR "practice pathway" OR algorithm OR "Guidance statement" OR "Guidance document" OR "Critical model" OR "clinical model" OR "practice model" OR "critical service model*" OR "clinical service model*" OR "practice service model" OR "model of care") OR abstract:("position statement" OR "position paper" OR "policy statement" OR "practice parameter" OR "best practice" OR standards OR guideline OR guidelines OR cpg OR cpgs OR consensus OR recommendations OR "critical path" OR "clinical path" OR "practice path" OR "critical pathway" OR "clinical pathway" OR "practice pathway" OR algorithm OR "Guidance statement" OR "Guidance document" OR "Critical model" OR "clinical model" OR "practice model" OR "critical service model*" OR "clinical service model*" OR "practice service model" OR "model of care"))) OR abstract:((title:("chronic primary pain" OR "chronic pain" OR "non-specific pain" OR "medically unexplained pain" OR "psychosomatic pain" OR "undiagnosed pain" OR "somatoform pain" OR "chronic nociceptive pain" OR "chronic nociplastic pain" OR "prolonged primary pain" OR "ongoing primary pain" OR "long standing primary pain" OR "longstanding primary pain" OR "enduring primary pain" OR "long lasting primary pain" OR "longlasting primary pain" OR "long-term primary pain" OR "longterm primary pain" OR "unrelenting primary pain" OR "un-relenting primary pain" OR "on-going primary pain" OR "persistent primary pain" OR "persisting primary pain" OR "recurrent primary pain" OR "constant primary pain" OR "sustained primary pain" OR "relentless primary pain" OR "drawn-out primary pain" OR "continuous primary pain" OR "ever-present primary pain" OR "non-remitting primary pain" OR "perpetual primary pain" OR "lingering primary pain" OR "protracted primary pain" OR "episodic primary pain*") OR abstract:("chronic primary pain" OR "chronic pain" OR "non-specific pain" OR "medically unexplained pain" OR "psychosomatic pain" OR "undiagnosed pain" OR "somatoform pain" OR "chronic nociceptive pain" OR "chronic nociplastic pain" OR "prolonged primary pain" OR "ongoing primary pain" OR "long standing primary pain" OR "longstanding primary pain" OR "enduring primary pain" OR "long lasting primary pain" OR "longlasting primary pain" OR "long-term primary pain" OR "longterm primary pain" OR "unrelenting primary pain" OR "un-relenting primary pain" OR "on-going primary pain" OR "persistent primary pain" OR "persisting primary pain" OR "recurrent primary pain" OR "constant primary pain" OR "sustained primary pain" OR "relentless primary pain" OR "drawn-out primary pain" OR "continuous primary pain" OR "ever-present primary pain" OR "non-remitting primary pain" OR "perpetual primary pain" OR "lingering primary pain" OR "protracted primary pain" OR "episodic primary pain*")) AND (title:("position statement" OR "position paper" OR "policy statement" OR "practice parameter" OR "best practice" OR standards OR guideline OR guidelines OR cpg OR cpgs OR consensus OR recommendations OR "critical path" OR "clinical path" OR "practice path" OR "critical pathway" OR "clinical pathway" OR "practice pathway" OR algorithm OR "Guidance statement" OR "Guidance document" OR "Critical model" OR "clinical model" OR "practice model" OR "critical service model*" OR "clinical service model*" OR "practice service model" OR "model of care") OR abstract:("position statement" OR "position paper" OR "policy statement" OR "practice parameter" OR "best practice" OR standards OR guideline OR guidelines OR cpg OR cpgs OR consensus OR recommendations OR "critical path" OR "clinical path" OR "practice path" OR "critical pathway" OR "clinical pathway" OR "practice pathway" OR algorithm OR "Guidance statement" OR "Guidance document" OR "Critical model" OR "clinical model" OR "practice model" OR "critical service model*" OR "clinical service model*" OR "practice service model" OR "model of care"))))

**Equals Total: 1,676**

LIMITED TO 2015 TO 2025 EQUALS **1316 RESULTS**

## Guidelines clearinghouses and repositories

| **International guidelines clearinghouses and repositories*** | **National guidelines clearinghouses and repositories*** |
| --- | --- |
| ECRI Guidelines Trust (<https://guidelines.ecri.org/>) | US Agency for Healthcare Research and Quality (AHRQ) (<https://www.ahrq.gov/research/publications/search.html>);  (<https://effectivehealthcare.ahrq.gov/products/collections/treatments-for-chronic-pain>) |
| TRIP medical database (<https://www.tripdatabase.com/>) | National Institutes of Health (NIH) (<https://www.nih.gov/>)  Search terms: "clinical practice guidelines", "practice guidelines" in  1. National Institute on Aging  2. National institute of arthritis and musculoskeletal and skin diseases  3. National institute of general medical sciences  4. Eunice Kennedy Shriver National Institute of Child Health and Human Development (NICHD)  Search whole website: "chronic pain"; "chronic primary pain" |
| BIGG, the international database of GRADE Guidelines (<https://bigg.bvsalud.org/en/home-en/>) | US Alliance for the Implementation of Clinical Practice Guidelines (<https://aicpg.org/ngc-summaries/>) |
| Guidelinecental (<https://www.guidelinecentral.com/guidelines/>) | eGuidelines UK (<https://eguidelines.co.uk>)  Search: 'Musculoskeletal and Joints' disease area hub |
| Guidelines International Network (<https://guidelines.ebmportal.com/>; <https://g-i-n.net>) | United Kingdom’s National Institute for Health and Clinical Excellence (NICE) (<https://www.nice.org.uk/about/what-we-do/our-programmes/nice-guidance/nice-guidelines>) |
| World Health Organization Guidelines repository (<https://www.who.int/publications/i?publishingoffices=c09761c0-ab8e-4cfa-9744-99509c4d306b>) | Scottish Intercollegiate Guidelines (SIGN) (<https://www.sign.ac.uk/our-guidelines/>) |
| MAGIC Evidence Ecosystem Foundation (<https://www.magicevidence.org/>) | Australian National Health and Medical Research Council (NHMRC) (<https://www.nhmrc.gov.au/guidelines>) |

*Search terms unless otherwise specified: “pain”; "chronic pain"; “chronic primary pain”; "primary pain"; "chronic primary".

## Pain care priority categories of the *“Listen to me, learn from me”* framework

Summative descriptions for each of the nine pain care priority categories, reproduced from Slater et al^18^ under licence agreement 6026271069724 provided by Wolters Kluwer Health, Inc. and Copyright Clearance Center.

| **Category** | **Summative description** |
| --- | --- |
| **Category 1: Validating, acknowledging and respecting each individual person’s pain experience** | Pain priorities reflect the need for health professionals to validate people’s individual pain experiences, most notably acknowledging and believing what a person is telling them about their pain (including their symptoms, history, impact and experiences); respecting their knowledge and lived experience and showing empathy. |
| **Category 2: Communication styles and consultation spaces to ensure safe, respectful and effective communication between health professionals and people living with chronic pain** | Priorities focus on communication interactions between health professionals and people living with pain.  Communication styles: People living with chronic pain and carers placed importance on health professionals: i) actively listening to hear their concerns; ii) learning from those living with chronic pain; and iii) responding appropriately to indicate their understanding of the impact of pain on the person. In discussing their chronic pain, people wanted health professionals to provide encouragement and hope where possible, give meaningful answers specific to their situation (using simple language), and to be honest with them if they don’t have an answer or can’t provide a diagnosis or reason for the pain.  Communication environments: People living with pain highlighted the importance of health professionals creating a safe consultation space for them to share opinions, ask questions and discuss relevant information without judgement. |
| **Category 3: Multidisciplinary team approach to pain care** | Health professionals working together to effectively coordinate care and ensure people living with chronic pain receive the right treatment at the right time is the key priority within this category. This includes timely referrals to medical specialists and allied health professionals to support pain management and functional ability, as well as receiving appropriate support services for mental health. |
| **Category 4: Holistic approach to pain care** | People emphasised the importance of health professionals adopting a holistic and tailored care approach that incorporates physical, mental, occupational, social, spiritual and intellectual needs, as well as other health issues, when managing chronic pain. It was also highlighted that health professionals need to demonstrate greater understanding of the complex nature of chronic pain. |
| **Category 5: Ensuring genuine partnership approaches in pain care** | A genuine partnership approach between health professionals and people living with chronic pain when discussing available treatment options, developing an appropriate management plan and subsequent monitoring was the overarching priority for this category. People stressed the need to consider contextual factors such as financial circumstances, geographic barriers and individual experiences and preferences when discussing pain management options. People living with pain also emphasised the importance of management strategies that supported their independence and focused on their quality of life. Explanations of risks and benefits for both pharmacologic and non-pharmacologic treatments and flexibility with treatment and care plans, rather than a ‘one size fits all’ approach were also considered important. |
| **Category 6: Knowledge and experience of health professionals to support pain care** | People highlighted the critical need for health professionals to be knowledgeable about different types of pain and to able to demonstrate an understanding of why pain can persist. Furthermore, people living with chronic pain advocated for health professionals to be willing to undertake research or consult more broadly across other health disciplines to better understand specific pain conditions, where required/appropriate. |
| **Category 7: Supportive self-management** | Key priorities focus on people with chronic pain being supported to self-manage their overall health. Support includes being directed to relevant evidence-based pain management resources (digital and non-digital); being provided with practical strategies applicable to day-to-day living; and, being able to access patient support and advocacy groups. |
| **Category 8: Safe use of medicines in pain care** | Accessing medications without stigma; supporting people living with pain in their choice of medication based on their preferences and experience; and prescribing safe medications to assist active participation in day-to-day living were key priorities. Several people with chronic pain also advocated for autonomy to adjust medication dosages based on pain levels. |
| **Category 9: Diagnosis/looking for a cause of pain** | The priorities here focus on health professionals providing a clear outline of how a person’s chronic pain will be investigated, as well as continuing to seek a diagnosis or reason for pain when tests or scans are inconclusive. People with chronic pain also highlighted the importance of health professionals looking for causes of pain without stigmatising people or being dismissive. |
