# Supplementary material for: Clinical practice guidelines for the care of people experiencing chronic primary pain: protocol for a systematic review with interpretation against an established chronic pain care priority framework
Source: BMJ Open. 2025 Sep 18;15(9):e105315. doi: 10.1136/bmjopen-2025-105315 (PMC12458848; doi:10.1136/bmjopen-2025-105315)
Supplement: Supplementary file 2 [file bmjopen-2025-105315.reviewer_comments.pdf]

PEER REVIEW HISTORY

BMJ Open publishes all reviews undertaken for accepted manuscripts. Reviewers are asked to complete a checklist review form and are provided with free text boxes to elaborate on their assessment. These free text comments are reproduced below.

ARTICLE DETAILS

Title (Provisional)

Clinical practice guidelines for the care of people experiencing chronic primary pain: Protocol for a systematic review with interpretation against an established chronic pain care priority framework

Authors

Briggs, Andrew M; Siegfried, Nandi; Waller, Robert; O'Connell, Neil E; Romero, Lorena; Klem, Nardia-Rose; Ampiah, Paapa Kwesi; Belton, Joletta L; Blyth, Fiona; De Morgan, Simone; Lord, Susan M; Nicholas, Michael; O'Sullivan, P; Shakya, Asim; Smith, Anne; Slater, Helen

---

VERSION 1 - REVIEW

Reviewer **1**

Name **Gleadhill, Connor**

Affiliation **The University of Newcastle, School of Medicine and Public Health**

Date **03-Jul-2025**

COI **None**

I congratulate the authors on an important paper. Publication of this protocol will bring awareness, transparency, trust, and rigour to a key study. I commend the authors' approach to improve the relevance of future Clinical Practice Guidelines (CPGs) for people living with chronic pain; CPGs are a key mechanism to better pain outcomes. I recommend some minor additions to the manuscript.

1. Methods, General comment:

The authors should be commended for their gold-standard approach to developing their research with patient research partners. The Advisory Group is another uniquely positive aspect of this study. Some more information about the make-up and function of end-user involvement in the Advisory Group will enhance the reader's understanding of the authors' approach. For example, who will make up the Advisory Group (people with lived experience of pain, professionals, researchers, policy and guideline developers)? At what points will the Advisory Group be involved - only at Stage 1, part 4? Or at multiple points during the

analysis and interpretation? Is the Advisory Group's function to broadly contextualise and make sense of the findings or to shape the interpretation during Stage 1 - 3? Does the Advisory Group have the remit to agree or disagree with the plotting of CPG recommendation data points on the matrix? Some of this information - about how the Advisory Group will shape the findings - could be made explicit when the Advisory Group is first mentioned (page 17, line 9).

Other information about who is part of the Advisory Group, and when they will be involved, could be summarised within the Patient and Public Involvement section.

2. Methods and analysis, Selection criteria, Table 1, page 9, line 53-4:

More information about the rationale behind excluding hospital inpatient or other institutionalised populations would be helpful. Presumably there is an important reason for this exclusion surrounding the care context and review objectives; the reader would benefit from more understanding on this.

3. Methods and analysis, Quality appraisal, Stage 3, page 19, line 29-31:

It would be helpful to have some more information about how authors will map the CPG content against the Listen to me, learn from me, framework. Will the recommendations that have been inputted into the described matrix and then interpreted be further ranked - alongside the other CPG text excerpts - against the framework?

**Reviewer**	**2**
**Name**	**van Rysewyk, Simon**
**Affiliation**	**University of Tasmania**
**Date**	**08-Aug-2025**
**COI**	**None**

Dear authors,

This is a clearly written and well-structured study protocol addressing a critical gap in chronic pain management. The authors have articulated the rationale and objectives with clarity, and the protocol is well-aligned with contemporary definitions of chronic primary pain under ICD-11. The systematic review methodology is robust, with comprehensive database and grey literature searches, and thoughtful use of the AGREE II and AGREE-REX appraisal tools.

The integration of the “Listen to me, learn from me” framework enhances the person-centred orientation of the review. Minor strengths include strong transparency in reporting, inclusion of lived experience partners, and a detailed explanation of inclusion/exclusion criteria.

One optional suggestion is to clarify how cultural differences in guideline development could affect mapping to the person-centred framework derived from Australian participants.

Overall, the manuscript meets high scientific and ethical standards for protocol publication and requires no major revisions.

Sincerely,

Dr Simon van Rysewyk

VERSION 1 - AUTHOR RESPONSE

<u>Reviewer: 1</u> Dr. Connor Gleadhill, The University of Newcastle, Hunter New England Population Health

I congratulate the authors on an important paper. Publication of this protocol will bring awareness, transparency, trust, and rigour to a key study. I commend the authors' approach to improve the relevance of future Clinical Practice Guidelines (CPGs) for people living with chronic pain; CPGs are a key mechanism to better pain outcomes. I recommend some minor additions to the manuscript.

1. Methods, General comment: The authors should be commended for their gold-standard approach to developing their research with patient research partners. The Advisory Group is another uniquely positive aspect of this study. Some more information about the make-up and function of end-user involvement in the Advisory Group will enhance the reader's understanding of the authors' approach. For example, who will make up the Advisory Group (people with lived experience of pain, professionals, researchers, policy and guideline developers)? At what points will the Advisory Group be involved - only at Stage 1, part 4? Or at multiple points during the analysis and interpretation? Is the Advisory Group's function to broadly contextualise and make sense of the findings or to shape the interpretation during Stage 1 - 3? Does the Advisory Group have the remit to agree or disagree with the plotting of CPG recommendation data points on the matrix? Some of this information - about how the Advisory Group will shape the findings - could be made explicit when the Advisory Group is first mentioned (page 17, line 9).

   Other information about who is part of the Advisory Group, and when they will be involved, could be summarised within the Patient and Public Involvement section.

   <u>*Authors' response*</u>*: Thank you for raising this important query. We agree with the Reviewer that this information is important and relevant to the review methods. As suggested, we have revised the text to signpost to readers to refer to the "Patient and Public Involvement" section for more information. In this subsection, we have expanded the commentary to include further details about the Advisory Group composition and outline at which stages of the review the members will be consulted. In addition, we have now explicitly mentioned in the Methods at what points in the review life course the Advisory Group members will be involved (i.e. stages 1-4 of the 'Data synthesis and judgement of certainty').*

2. Methods and analysis, Selection criteria, Table 1, page 9, line 53-4:
   More information about the rationale behind excluding hospital inpatient or other institutionalised populations would be helpful. Presumably there is an important reason for this exclusion surrounding the care context and review objectives; the reader would benefit from more understanding on this.

   Authors' response: *Thank you for this query about the criteria for "Population and clinical indication(s) and condition(s)". We excluded hospitalised inpatients and other institutionalised populations (e.g. people who are incarcerated) since care delivery in these settings (including interventions delivered, access to workforce and access to multimodal interventions) would vary considerably from populations outside these settings, such as in primary and community care and care in residential aged care facilities, where the majority of people receiving care for chronic primary pain would be located. We consider that inpatients and other institutionalised populations represent discrete and likely more homogenous populations with specific care needs that would differ substantially to people in primary, community and residential aged care settings. In this context, it would not be clinically meaningful or helpful to combine recommendations for care with other (non-institutionalised) populations. We have added a summary of this response to the PICAR framework described under "Selection criteria", as recommended by the Reviewer.*

3. Methods and analysis, Quality appraisal, Stage 3, page 19, line 29-31:
   It would be helpful to have some more information about how authors will map the CPG content against the Listen to me, learn from me, framework. Will the recommendations that have been inputted into the described matrix and then interpreted be further ranked - alongside the other CPG text excerpts - against the framework?

   Authors' response: *Thank you for requesting further clarification about the proposed mapping approach. We will map two sets of data against the nine categories of the "Listen to me, learn from me" framework. First, we will identify and map, which, if any, of the extracted CPG recommendations (from stage 1 analysis) align with one of more of the nine framework categories. Alignment will be determined by considering the verbatim recommendation against the detailed description of each category from the "Listen to me, learn from me" framework (as outlined in the table in the supplementary file). Where alignment between a CPG recommendation and a framework category is equivocal, we will interrogate the CPG recommendation further against the specific granular items underpinning that framework category (i.e. there are 52 items underpinning the 9 categories of the framework)[1]. Second, we will repeat this process using the findings from the stage 2 analysis in which we synthesise CPG text excerpts, other than formal CPG recommendations. At the end of this mapping process, we will create a grid or heat map (or similar, depending on the nature of the data) that summarises the extent to which person-centred pain care priorities are explicitly reflected in the included CPGs.*

   This information has now been added to the revised manuscript in the Stage 3 analysis section.

__Reviewer: 2__ **Dr. Simon van Rysewyk, University of Tasmania**

Dear authors, this is a clearly written and well-structured study protocol addressing a critical gap in chronic pain management. The authors have articulated the rationale and objectives with clarity, and the protocol is well-aligned with contemporary definitions of chronic primary pain under ICD-11. The systematic review methodology is robust, with comprehensive database and grey literature searches, and thoughtful use of the AGREE II and AGREE-REX appraisal tools.
The integration of the "Listen to me, learn from me" framework enhances the person-centred orientation of the review. Minor strengths include strong transparency in reporting, inclusion of lived experience partners, and a detailed explanation of inclusion/exclusion criteria.
Overall, the manuscript meets high scientific and ethical standards for protocol publication and requires no major revisions.

1. One optional suggestion is to clarify how cultural differences in guideline development could affect mapping to the person-centred framework derived from Australian participants.

   __Authors' response__: Thank you for raising this important point. We appreciate the Reviewer's consideration that using an Australian-derived framework to map against international guidelines may reduce confidence in the findings. While we acknowledge the "Listen to me, learn from me" framework was empirically derived from people with lived pain experience in Australia, we consider that the priority domains, from the perspective of a person-centered approach to chronic primary pain care, would be largely relevant across cultures. As a further strategy to consider cross-cultural and cross-context considerations in CPG development and CPG alignment with the "Listen to me, learn from me" framework, we have intentionally assembled a diverse international review team with representation across high-income and low- and middle-income settings, gender, professional background and life course stage. As such, we feel the Advisory Group is well placed to provide appropriate input into any cross-cultural considerations in CPG development and importantly, contribute to how we collectively interpret the outcomes of the stage 3 analysis described in the protocol.

References

1. Slater H, Jordan JE, O'Sullivan PB, et al. 'Listen to me, learn from me': a priority setting partnership for shaping interdisciplinary pain training to strengthen chronic pain care. *Pain* 2022; **163**(11): 1145-63.
